# Biochemical and hematological reference intervals in rhesus and cynomolgus macaques and implications for vaccine and drug development

**DOI:** 10.1038/s41684-025-01547-y

**Published:** 2025-05-16

**Authors:** Xianglei Yan, Rodrigo Arcoverde Cerveira, Sebastian Ols, Klara Lenart, Fredrika Hellgren, Marcos Miranda, Olivia Engstrand, Annika Reinhardt, Bengt Eriksson, Karin Loré

**Affiliations:** 1 Division of Immunology and Respiratory Medicine, Department of Medicine Solna, Karolinska Institutet and Karolinska University Hospital, Stockholm, Sweden; 2 Center of Molecular Medicine, Stockholm, Sweden; 3 Astrid Fagraeus Laboratory, Comparative Medicine, Karolinska Institutet, Stockholm, Sweden; 4 Present Address: Institute for Protein Design, University of Washington, Seattle, WA USA

**Keywords:** Immunological models, Vaccines, Animal physiology, Biochemical assays, Drug safety

## Abstract

Nonhuman primates have a key role in the evaluation of novel therapeutics including vaccine and drug development. Monitoring biochemical and hematological parameters of macaques is critical to understand toxicity and safety, but general reference intervals following standardized guidelines remain to be determined. Here we compiled multiple internal datasets to define normal ranges of classical biochemical and hematological parameters in Indian and Chinese rhesus macaques as well as cynomolgus macaques. Furthermore, the combination of hematological data with phenotypic information of cells obtained by flow cytometry enabled analyses of specific immune cell subsets. We found that vaccination generally induced transient changes at 24 h in cell frequencies accompanied by fluctuation in selected liver enzymes and metabolites. However, most parameters remained within our identified reference intervals. These deviations did not lead to noticeable side effects. Fluctuation in selected biochemical and hematological parameters was accompanied with differentiation of CD14^+^CD16^+^ intermediate monocytes and upregulation of genes associated with interleukin-1 signaling. By contrast, two animals with noticeable side effects showed sustained deviations. This study provides insights into baseline and vaccine-induced biochemical and hematological profiles of healthy macaques, facilitating the interpretation of toxicity and safety assessments in preclinical trials of novel therapies.

## Abstract (web summary)

This study, which establishes reference intervals for biochemical and hematological parameters in healthy macaques and describes changes in these parameters after vaccination, will facilitate toxicity monitoring in preclinical vaccine studies.

## Main

In the preclinical phase of drug development including vaccine testing, in vivo studies are usually first performed in mouse models. However, data from mice may not accurately reflect responses in humans^1,2^. The mouse strains typically used in immunological studies are inbred^1,3^. There are also clear differences between mice and humans in terms of the composition of immune cell populations and expression of immune receptors^4^. By contrast, nonhuman primates (NHPs) such as rhesus macaques (*Macaca mulatta*) and cynomolgus macaques (*Macaca fascicularis*) are highly similar to humans in terms of anatomy, physiology and genetic diversity. Humans share about 93% of genome identity with macaques^5,6^, while there is 66% similarity with mice^6,7^. In vaccine and drug development, NHPs are therefore particularly valuable models for testing safety and pharmacokinetics before entering clinical trials. The critical value of NHPs as preclinical animal models was evident in the development of SARS-CoV-2 vaccines during the pandemic^8,9^. However, this has resulted in a current worldwide shortage of laboratory NHPs^9,10^, underscoring the necessity of careful planning and design of studies to obtain sufficient data from the minimal number of animals when using this model.

Rhesus macaques, classified as Old-World monkeys, are widely distributed in Asia mainly in India and southern China. Based on their country of origin, they are divided into Indian-derived and Chinese-derived rhesus macaques, each with two to four respective subspecies^11,12^. Genetically, Indian-derived and Chinese-derived rhesus macaques exhibit a high degree of allelic overlap^13^, indicating shared genetics, although there is substantial genetic diversity between these two subgroups due to distinct geographical and evolutionary histories^13^. Notably, even within the same subgroup, there can be subtle genetic variations between individuals from different regions^5^.

The cynomolgus macaque, also known as the long-tailed macaque or crab-eating macaque, is another species in the *Macaca* genus found in Southeast Asia. Cynomolgus macaques are more genetically homogeneous than rhesus macaques owing to their more restricted distribution. Although they share some conserved alleles with rhesus macaques, cynomolgus macaques exhibit a distinct set of genetic variations^13^. This diversity extends to their immune system, potentially influencing their responses to pathogens, vaccines and other immunological challenges. The genetic diversity of macaques is particularly valuable in immunological studies to mimic human diversity.

Evaluation of standard biochemical and hematological parameters in blood has a central role in monitoring toxicity during treatment^14–18^. Macaques and humans share several biochemical and hematological parameters^19–21^. However, unlike in humans, normal ranges of these parameters have not been definitively determined and widely adopted in macaques^22–28^. In addition, variables such as species, subgroup, age, sex, diet, living environment and methods used for assessments have also been reported to influence these parameters^22–28^. More studies to identify reference intervals (RIs) in macaques are warranted and would be important for distinguishing true pathological effects in treatment studies. In this study, we analyzed and compiled multiple data from various vaccine studies performed in healthy rhesus and cynomolgus macaques at the Karolinska Institutet (KI), Sweden, to calculate internal RIs of several classical parameters in each species and in the age range commonly applicable for vaccine studies. We compared the data with the large Primate Aging Database (PAD). Furthermore, we evaluated how these parameters were altered in animals with or without substantial adverse effects and innate immune activation early after vaccination.

## Results

### Age differences in biochemical and hematological parameters

The objective of this study was to define internal RIs of classical biochemical and hematological parameters in healthy macaques housed at KI and sampled longitudinally. The animals included both Indian (*n* = 77, 41 male (M) and 36 female (F)) and Chinese (*n* = 36, 18 M and 18 F) rhesus macaques as well as cynomolgus macaques (*n* = 22, 10 M and 12 F). Datasets for Indian rhesus macaques met the requirements to achieve 80% (1 − *β* = 0.8) statistical power for variables such as age, sex and social hierarchy, based on a medium effect size (Cohen’s *d* = 0.5 or Cohen’s *f* = 0.25) and a significance level of 0.05. Because these are relatively small datasets, we used data from PAD, a publicly accessible repository supported by the National Institutes of Health as reference. In animal studies conducted at KI, Indian rhesus macaques often consist of young adults (3–7 years) (Table 1). To determine whether there are differences between young adults and other age groups, we first used the data from PAD. PAD comprises diverse biological data collected across multiple NHP facilities. The PAD datasets include metrics such as blood chemistry, hematology and body weight of NHPs in captivity, in good health and of different ages.

**Table 1:** Information on NHP studies sourced for this study^43–47^, including unpublished ongoing studies

Year study performed	Species and sample size	Age range (years)	Vaccine types	Administration route	Reference
2016	Indian *Macaca mulatta*, *n* = 24 (14 M + 10 F)	M: 2.7–4.2, F: 2.7–3.7	Protein vaccine in adjuvant	s.c. or i.m.	Thompson et al.^45^
2017	Indian *Macaca mulatta*, *n* = 14 (4 M + 10 F)	M: 4.0–4.3, F: 3.8–4.8	Protein vaccine in adjuvant	i.m.	Ols et al.^46^
2018	Chinese *Macaca mulatta*, *n* = 18 (9 M + 9 F)	M: 3.2–3.5, F: 3.1–3.5	mRNA vaccine or inactivated virus vaccine	i.m.	Hellgren et al.^43^
2018	Chinese *Macaca mulatta*, *n* = 18 (9 M + 9 F)	M: 3.4–3.7, F: 3.3–3.8	mRNA vaccine	i.m.	Not yet published
2020–2021	Indian *Macaca mulatta*, *n* = 12 (6 M + 6 F)	M: 4.3–6.8, F: 4.4–6.3	Protein vaccine in adjuvant	i.m.	Lenart et al.^44^
2020–2021	Indian *Macaca mulatta*, *n* = 12 (9 M + 3 F)	M: 4.4–6.2, F: 5.1–5.2	Peptides with anti-CD40 mAb	s.c. or i.v.	Yan et al.^47^
2023	Indian *Macaca mulatta*, *n* = 15 (8 M + 7 F)	M: 7.4–9.3, F: 7.4–9.4	mRNA vaccine	i.m.	Not yet published
2023	*Macaca fascicularis*, *n* = 20 (10 M + 10 F)	M: 4.2–4.5, F: 3.9–6.4	Protein vaccine in adjuvant	i.m.	Not yet published
2023	*Macaca fascicularis*, *n* = 2 (2 F)	F: 11.5–16.8	Peptides with anti-CD40 mAb	s.c.	Not yet published

i.m., intramuscular injection; i.v., intravascular injection; mAb, monoclonal antibody; s.c., subcutaneous injection.

Classical biochemical parameters available for macaques include leakage enzymes such as alanine transaminase (ALT), induced enzymes such as alkaline phosphatase (ALP), γ-glutamyl transferase (GGT) and liver function markers including bile acid (BA), total bilirubin (TBIL), albumin (ALB), cholesterol (CHOL) and blood urea nitrogen (BUN) (Table 2). The liver is a critical organ for detoxification, bile secretion and protein production in mammals^29^. These parameters mainly indicate the status of the liver and kidneys and are widely monitored in vaccine and drug studies^29,30^. Furthermore, the immune system of macaques resembles that of humans, and thus similar hematological parameters can be measured^19–21^. We therefore analyzed clinically and routinely used hematological parameters (Table 3) such as hematocrit (HCT) and hemoglobin (HGB) for red blood cell mass, which together with red blood cell absolute count (RBC) assess red blood cell characteristics. Other parameters for measuring RBC quality included red cell distribution width-coefficient of variation (RDW%), standard deviation (RDWa), mean corpuscular volume (MCV), mean corpuscular HGB (MCH) and mean corpuscular HGB concentration (MCHC), which help to interpret anemia and other RBC disorders^31,32^. We also measured platelet absolute count (PLT) and mean platelet volume (MPV) for the number and size, respectively, to evaluate the production, consumption and destruction status of platelets^33^. Finally, white blood cell absolute count (WBC) as well as the subcomponents lymphocyte absolute count (LYM), granulocyte absolute count (GRAN) and monocyte absolute count (MONO) were analyzed to assess immunological changes^34–36^.

**Table 2:** Classical biochemical parameters investigated in this study

Parameter	Abbreviation	Unit
**Liver**		
Alanine transaminase	ALT	U/L
Alkaline phosphatase	ALP	U/L
γ-Glutamyl transferase	GGT	U/L
Bile acid	BA	µmol/L
Total bilirubin	TBIL	µmol/L
Albumin	ALB	g/L
Cholesterol	CHOL	mmol/L
**Liver and kidney**		
Blood urea nitrogen	BUN	mmol/L

**Table 3:** Classical hematological parameters investigated in this study

Parameter	Abbreviation	Unit
**Red blood cells**		
Red blood cell count	RBC	×10^12^/L
Hematocrit	HCT	%
Hemoglobin	HGB	g/dL
Red cell distribution width, coefficient of variation	RDW%	%
Red cell distribution width, standard deviation	RDWa	fL
Mean corpuscular volume	MCV	fL
Mean corpuscular hemoglobin	MCH	pg
Mean corpuscular hemoglobin concentration	MCHC	g/dL
**Platelets**		
Platelet count	PLT	×10^9^/L
Mean platelet volume	MPV	fL
**White blood cells**		
White blood cell count	WBC	×10^9^/L
Lymphocyte count	LYM	×10^9^/L
Granulocyte count	GRAN	×10^9^/L
Monocyte count	MONO	×10^9^/L

**Fig. 1:**
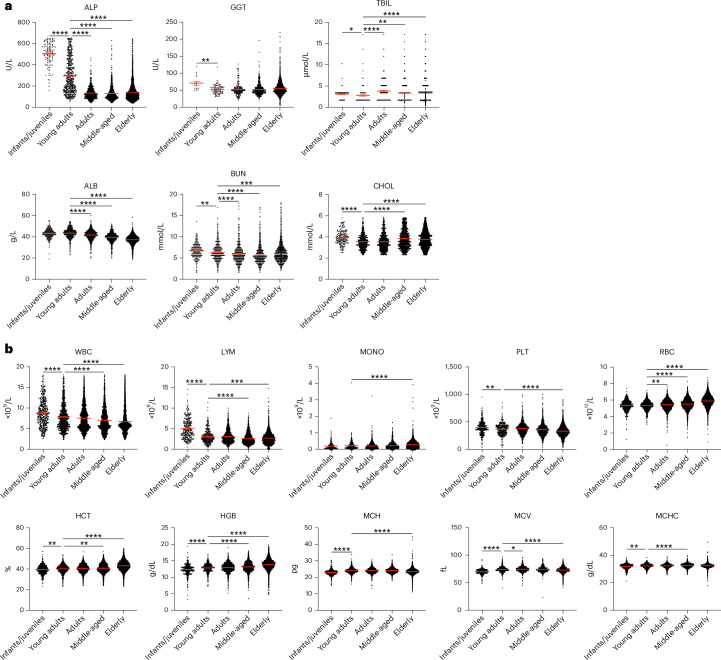
Age-based stratification of biochemical and hematological parameters across the lifespan of Indian rhesus macaques in the PAD. Datasets of healthy Indian rhesus macaques in captivity in the PAD were stratified into five groups: infants/juveniles (<3 years), young adults (3–7 years), adults (7–12 years), middle-aged (12–17 years) and elderly (≥17 years). The sample size (*n*) ranged from 76 to 5,824 per group. **a**, A comparison of biochemical parameters between different age groups. **b**, A comparison of hematological parameters between different age groups. A Kruskal–Wallis test with Dunn’s test corrected for multiple comparisons using statistical hypothesis testing was used. *P < 0.05, **P < 0.01, ***P < 0.001, ****P < 0.0001. The red lines indicate mean ± s.e.m.

We stratified the Indian rhesus macaque data obtained from PAD by age groups: infants/juveniles (<3 years), young adults (3–7 years), adults (7–12 years), middle-aged (12–17 years) and elderly (≥17 years). The age categories were based on previous similar studies^22,25^, as there are no standardized groupings for macaques owing to their gradual and variable aging process. A total of 76–5,824 data points were included in each age group (Supplementary Table 1). Most of the parameters became stable when animals reached adulthood, although we observed age-related differences in some parameters (Fig. 1a,b). Except for TBIL, most biochemical parameters showed decreased levels from the infant/juvenile ages to adulthood (Fig. 1a). ALP levels, known to correlate with growth, decreased with age, probably due to reduced growth rate and cessation of bone growth^26^.

For the hematological parameters, WBC, LYM and PLT levels decreased with age. MCH and MCV levels increased slightly with age, while MCHC decreased (Fig. 1b). These findings align well with human aging studies^37,38^. RBC, HCT, HGB and MONO levels increased with age in Indian rhesus macaques, contrary to human studies^37,38^. This underscores that using age-appropriate RIs may be important when evaluating experimental results and health.

### Comparisons of biochemical and hematological parameters of macaques at primate facilities

**Fig. 2:**
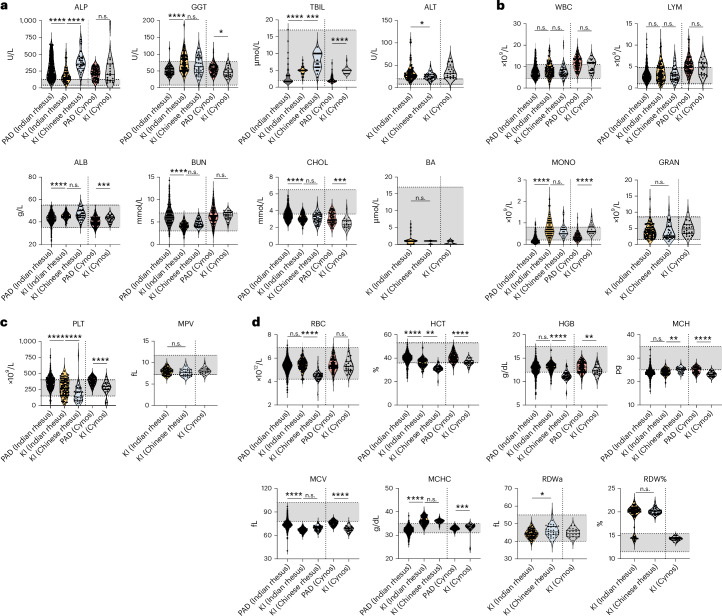
Comparison between same age-grouped NHPs housed at KI and those in the PAD. The samples size (*n*) ranged from 22 to 853 per group. **a**, Comparisons of biochemical parameters between Indian and Chinese rhesus macaques and cynomolgus macaques (Cynos) housed at KI and those in the PAD, if applicable. **b**–**d**, Comparisons of leukocyte-related (**b**), thrombocyte-related (**c**) and erythrocyte-related (**d**) hematological parameters between Indian and Chinese rhesus macaques and cynomolgus macaques housed at KI and those in the PAD, if applicable. The dashed lines and gray areas indicate human reference values. A Mann–Whitney *U* test and a Kruskal–Wallis test with Dunn’s test corrected for multiple comparisons using statistical hypothesis testing was used. *P < 0.05, **P < 0.01, ***P < 0.001, ****P < 0.0001. n.s., not significant. Lines in the violins indicate the median, the first quartile and the third quartile.

We conducted a comparison of available datasets from animals housed at the primate facility of KI with data obtained from the PAD. For most parameters in the similar young-adult age group, there was extensive overlap in the levels from PAD and the levels recorded at KI from Indian rhesus macaques (Fig. 2a–d), although some statistical differences were reached. Chinese rhesus macaques are not represented in PAD. However, we found that the Chinese rhesus macaques at KI displayed higher mean levels of ALP and TBIL compared with Indian rhesus macaques, although it is unclear if this has any biological relevance. For biochemical parameters, cynomolgus macaques at KI also showed levels mostly in line with those reported in PAD (Fig. 2a). As a point of reference, we compared the NHP data with the established RIs (gray area) for humans^14–18^. For parameters such as GGT, ALB and BUN, the data from macaques were within the human RIs. By contrast, there were consistently higher levels of ALP and ALT in macaques and lower values of CHOL compared with the normal range in humans (Fig. 2a).

In terms of hematological parameters, the macaques at KI had levels largely consistent with PAD (Fig. 2b–d). We observed higher MONO levels across all three macaque types at KI compared with PAD data (Fig. 2b). Notably, RBC, HCT and HGB levels in Chinese rhesus macaques were slightly lower than in Indian rhesus macaques but still within the ranges in PAD (Fig. 2d). Compared with human RIs^14–18^, macaques generally showed lower HCT, HGB, MCH and MCV parameters (Fig. 2d). This further emphasizes the need for species-specific RIs when interpreting laboratory results, and that human RIs cannot be applied indiscriminately. There can also be slight differences in parameters between macaques analyzed at different sites.

Some sex-based differences were observed in the biochemical and hematological parameters among Indian rhesus macaques (Supplementary Fig. 1a–c) as reported earlier^22–25^, probably due to physiological and hormonal variations. We did not find that social hierarchy significantly affected biochemical and hematological parameters in the Indian rhesus macaques at KI (Supplementary Fig. 2a–c).

### Establishment of internal RIs of biochemical and hematological parameters

To establish RIs that follow the EP28-A3c guideline^39^, which is a gold-standard approach used in clinical laboratories, and reflect a healthy range in macaques at KI, we compiled each internal datasets of Indian (*n* = 77, 41 M and 36 F) and Chinese (*n* = 36, 18 M and 18 F) rhesus macaques and cynomolgus macaques (*n* = 22, 10 M and 12 F). Each dataset included 8 biochemical parameters and 14 hematological parameters. The datasets were initially compiled by applying the robust regression and outlier removal (ROUT) test, with the maximum desired false discovery rate (*Q*) set to 1%, to identify outliers, followed by testing for normal distribution. Depending on the normality and sample size, two methods were used for analyzing the cleaned data: (A) if the data points were consistent with normal distribution, the upper limit was calculated as mean + 1.96 × s.d. and the lower limit as mean − 1.96 × s.d.; (B) if the data points were not consistent with normal distribution and the sample size was less than 120, the robust method described in the EP28-A3c guideline was used^39^. This method combines parametric and nonparametric approaches, using robust measures of location and spread instead of the mean and standard deviation^39,40^. This resulted in the calculated ranges of RIs, representing 95% of the data points, as reported in Table 4. Because we had more data available for Indian rhesus macaques, their RIs are probably more robust.

**Table 4:** Calculated ranges of RIs for biochemical and hematological parameters as per the EP28-A3c guideline for Indian and Chinese rhesus macaques (*Macaca mulatta*) and cynomolgus macaques (*Macaca fascicularis*)

Species	Parameter		Number of values	Mean	s.d.	Lower 95% CI	Upper 95% CI	Min	Max	2.5% percentile	97.5% percentile	RI	Unit
Indian *Macaca mulatta*	Biochemical	ALP	40	169.60	76.50	145.10	194.00	47.00	396.00	47.38	393.80	47.00–316.55	U/L
		ALT	80	29.56	10.08	27.32	31.81	12.00	62.00	12.08	61.72	7.63–48.45	U/L
		GGT	85	74.48	23.73	69.36	79.60	34.00	127.00	35.60	125.60	24.72–121.49	U/L
		BA	82	0.67	0.55	0.55	0.79	0.00	3.00	0.00	1.00	0.00–1.00	µmol/L
		TBIL	86	4.85	0.95	4.65	5.05	2.00	8.00	3.00	7.00	3.00–7.00	µmol/L
		ALB	86	45.44	2.73	44.86	46.03	38.00	52.00	39.00	51.83	40.09–50.79	g/L
		BUN	86	4.06	0.86	3.88	4.25	1.50	6.20	2.32	5.90	2.38–5.75	mmol/L
		CHOL	86	3.13	0.48	3.03	3.24	1.90	4.20	2.32	4.08	2.19–4.08	mmol/L
	Hematological	RBC	81	5.44	0.47	5.34	5.55	4.19	6.62	4.21	6.34	4.52–6.36	×10^12^/L
		WBC	81	8.57	3.40	7.82	9.32	1.70	18.50	3.01	17.49	1.24–14.97	×10^9^/L
		PLT	81	281.00	134.00	251.30	310.60	23.00	537.00	44.10	494.00	13.17–551.75	×10^9^/L
		GRAN	79	3.91	1.84	3.50	4.32	1.00	10.10	1.30	8.20	1.00–7.40	×10^9^/L
		LYM	81	3.71	2.14	3.24	4.18	0.30	10.40	0.71	9.69	0.30–7.80	×10^9^/L
		MONO	81	0.74	0.40	0.66	0.83	0.00	2.10	0.10	1.80	0.00–1.50	×10^9^/L
		HCT	76	36.03	3.01	35.34	36.71	28.40	41.50	28.86	40.95	30.06–42.37	%
		HGB	75	13.06	0.90	12.85	13.26	10.20	15.10	10.74	14.56	11.35–14.96	g/dL
		MCH	77	24.17	1.32	23.87	24.47	19.50	27.30	21.40	26.54	21.57–26.77	pg
		MCV	77	66.83	2.93	66.17	67.50	59.40	73.90	59.88	72.48	61.09–72.57	fL
		MCHC	77	36.18	1.70	35.79	36.56	32.80	39.50	32.90	39.22	32.69–39.62	g/dL
		RDWa	77	44.35	2.42	43.80	44.89	38.70	50.30	39.94	49.26	39.62–49.08	fL
		RDW%	62	20.37	0.71	20.19	20.55	18.90	22.20	18.96	22.20	18.98–21.76	%
		MPV	76	7.96	0.80	7.78	8.15	6.40	9.70	6.68	9.61	6.39–9.53	fL
Chinese *Macaca mulatta*	Biochemical	ALP	34	390.20	138.70	341.80	438.60	162.00	718.00	162.00	718.00	72.82–658.66	U/L
		ALT	34	26.44	7.56	23.80	29.08	13.00	48.00	13.00	48.00	11.63–41.25	U/L
		GGT	34	67.35	25.56	58.43	76.27	24.00	118.00	24.00	118.00	17.25–117.45	U/L
		BA	34	0.76	0.43	0.61	0.91	0.00	1.00	0.00	1.00	0.00–1.00	µmol/L
		TBIL	34	7.79	2.33	6.98	8.61	3.00	12.00	3.00	12.00	2.93–12.70	µmol/L
		ALB	34	47.09	4.49	45.52	48.65	38.00	56.00	38.00	56.00	38.29–55.89	g/L
		BUN	34	4.72	0.83	4.43	5.01	3.00	6.50	3.00	6.50	3.10–6.35	mmol/L
		CHOL	34	3.12	0.59	2.92	3.33	2.10	4.60	2.10	4.60	1.97–4.27	mmol/L
	Hematological	RBC	34	4.51	0.26	4.42	4.60	4.05	5.25	4.05	5.25	4.00–5.02	×10^12^/L
		WBC	35	7.47	2.16	6.73	8.21	3.30	11.90	3.30	11.90	3.24–11.69	×10^9^/L
		PLT	35	155.80	92.39	124.10	187.60	32.60	381.00	32.60	381.00	32.60–345.41	×10^9^/L
		GRAN	35	3.65	2.13	2.92	4.39	1.00	9.10	1.00	9.10	1.00–7.70	×10^9^/L
		LYM	36	3.28	1.66	2.71	3.84	0.80	7.10	0.80	7.10	0.80–6.31	×10^9^/L
		MONO	34	0.60	0.24	0.52	0.68	0.20	1.40	0.20	1.40	0.04–1.10	×10^9^/L
		HCT	35	31.59	2.08	30.88	32.31	28.20	37.00	28.20	37.00	27.52–35.66	%
		HGB	35	11.34	0.72	11.09	11.58	10.40	13.30	10.40	13.30	9.69–12.69	g/dL
		MCH	36	25.00	1.09	24.63	25.37	22.80	27.40	22.80	27.40	22.87–27.13	pg
		MCV	36	69.45	3.20	68.37	70.53	62.70	76.80	62.70	76.80	63.17–75.73	fL
		MCHC	36	36.01	0.80	35.73	36.28	33.60	37.90	33.60	37.90	34.44–37.58	g/dL
		RDWa	36	45.92	3.34	44.79	47.05	39.90	55.80	39.90	55.80	39.38–52.46	fL
		RDW%	35	20.15	0.68	19.92	20.39	18.80	22.10	18.80	22.10	18.82–21.48	%
		MPV	35	7.71	0.82	7.42	7.99	6.40	9.50	6.40	9.50	6.09–9.32	fL
*Macaca fascicularis*	Biochemical	ALP	20	223.30	122.50	165.90	280.60	78.00	448.00	78.00	448.00	78.00–463.40	U/L
		ALT	21	35.57	14.07	29.17	41.98	20.00	65.00	20.00	65.00	2.40–64.66	U/L
		GGT	21	47.38	14.70	40.69	54.07	25.00	74.00	25.00	74.00	18.57–76.19	U/L
		BA	21	0.33	0.48	0.11	0.55	0.00	1.00	0.00	1.00	0.00–1.00	µmol/L
		TBIL	21	4.91	0.94	4.48	5.33	4.00	8.00	4.00	8.00	4.00–8.00	µmol/L
		ALB	21	43.19	2.73	41.95	44.43	38.00	47.00	38.00	47.00	37.84–48.54	g/L
		BUN	21	6.45	0.86	6.06	6.84	4.50	7.60	4.50	7.60	4.77–8.14	mmol/L
		CHOL	21	2.52	0.59	2.25	2.79	1.60	4.20	1.60	4.20	1.37–3.67	mmol/L
	Hematological	RBC	22	5.34	0.52	5.11	5.57	4.58	6.56	4.58	6.56	4.32–6.37	×10^12^/L
		WBC	22	10.53	2.63	9.37	11.70	5.90	15.10	5.90	15.10	5.39–15.68	×10^9^/L
		PLT	22	272.20	108.70	224.00	320.40	33.00	447.00	33.00	447.00	67.18–531.84	×10^9^/L
		GRAN	22	4.94	2.09	4.01	5.87	1.70	10.00	1.70	10.00	0.83–9.04	×10^9^/L
		LYM	22	4.88	1.72	4.12	5.65	2.40	8.60	2.40	8.60	1.50–8.26	×10^9^/L
		MONO	22	0.71	0.22	0.61	0.81	0.40	1.30	0.40	1.30	0.12–1.18	×10^9^/L
		HCT	22	36.55	2.52	35.43	37.67	31.50	40.90	31.50	40.90	31.60–41.50	%
		HGB	22	12.33	0.99	11.89	12.77	10.40	14.30	10.40	14.30	10.38–14.28	g/dL
		MCH	22	23.16	0.99	22.72	23.60	21.50	24.90	21.50	24.90	21.22–25.10	pg
		MCV	22	68.66	3.65	67.04	70.28	62.30	76.30	62.30	76.30	61.50–75.82	fL
		MCHC	21	33.75	0.78	33.40	34.11	32.20	35.10	32.20	35.10	32.23–35.27	g/dL
		RDWa	22	44.79	2.30	43.77	45.80	40.30	49.00	40.30	49.00	40.29–49.29	fL
		RDW%	22	14.27	0.40	14.10	14.45	13.50	15.00	13.50	15.00	13.49–15.05	%
		MPV	21	8.05	0.70	7.73	8.37	6.80	9.80	6.80	9.80	6.67–9.42	fL

RI ranges are presented per the EP28-A3c guideline^39^. CI, confidence interval.

### Transient biochemical and hematological changes observed after immunization

**Fig. 3:**
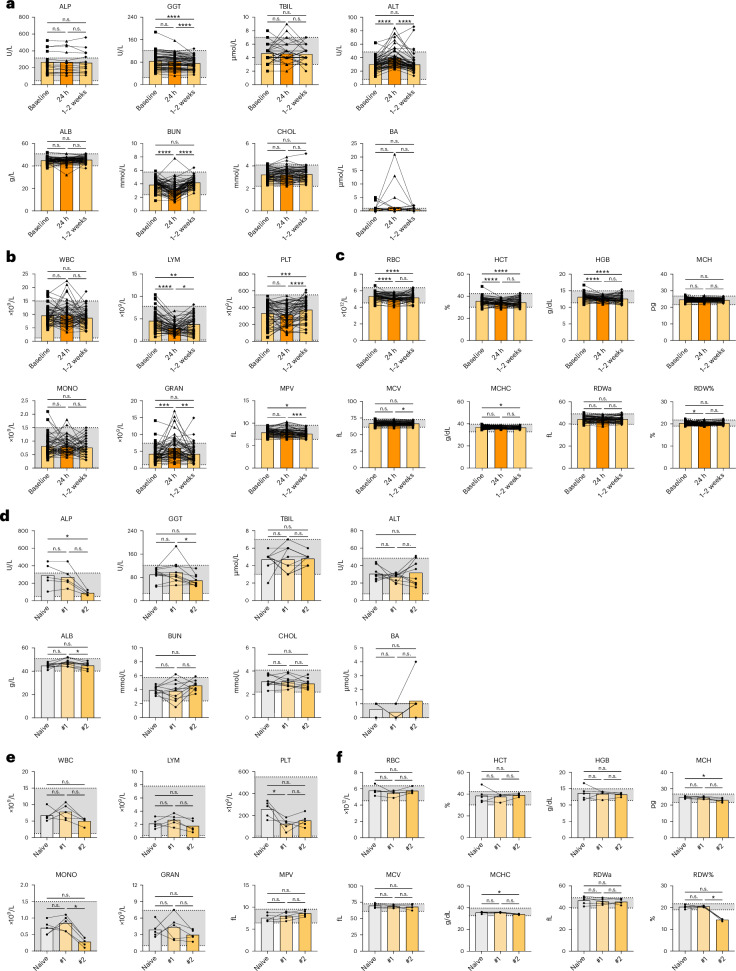
Comparison of biochemical and hematological parameters in Indian rhesus macaques before and after immunization in four protein vaccine studies^44–47^. The vaccine studies are listed in Table 1. Samples sizes: *n* = 59 (for biochemical parameters) or 54 (for hematological parameters). **a**–**c**, Peripheral blood samples were collected from Indian rhesus macaques at baseline, 24 h and 1–2 weeks after immunization, and biochemical and hematological parameters were measured. Results are shown as comparisons of biochemical parameters (**a**), leukocyte-related and thrombocyte-related (**b**), and erythrocyte-related (**c**) hematological parameters. Symbols (squares, triangles and diamonds) represent data points from different time points from the same animals, and the bars indicate the mean values. **d**–**f**, Longitudinal analysis of baseline samples after recurrent vaccine administrations. Naive, animals with no prior experience of vaccination; #1, animals that experienced one vaccine study, approximately 1 year after the first vaccination; #2, animals that experienced a second vaccine study, approximately 3 years after the first vaccination. Results are shown as comparisons of biochemical parameters (**d**), leukocyte-related and thrombocyte-related (**e**), and erythrocyte-related (**f**) hematological parameters. The bars indicate the mean values. The dashed lines and gray areas indicate the identified Indian rhesus macaque RIs. A Friedman test with Dunn’s test corrected for multiple comparisons was used. *P < 0.05, **P < 0.01, ***P < 0.001, ****P < 0.0001. n.s., not significant.

We and others have demonstrated that several types of vaccines induce a rapid inflammatory response with short duration^41–44^. We applied the RIs to evaluate whether vaccination induced detectable changes in biochemical and hematological parameters. We compiled data from multiple studies on macaques before and 24 h after receiving vaccination (Table 1). The vaccines included protein or peptides in adjuvant or mRNA vaccines. All the animals in these studies induced well-detectable immune responses after vaccination but none^43–46^ exhibited visible adverse effects except for two^47^, which were further investigated later. We primarily focused on the studies using Indian rhesus macaques because there were more data available from this population. We found no statistically significant changes in the levels of ALP, TBIL, ALB, CHOL and BA 24 h after immunization compared with baseline values (Fig. 3a). ALT increased significantly (Friedman test, *P* < 0.0001), while BUN decreased significantly (Friedman test, *P* < 0.0001) 24 h after immunization; however, both parameters returned to baseline levels within 1–2 weeks. GGT did not change significantly at 24 h after immunization but showed a slight decrease at 1–2 weeks (Fig. 3a). These results demonstrate that there is a transient and mild effect on the liver by vaccination, which usually recovers within 1–2 weeks.

In terms of hematological parameters, LYM decreased in peripheral blood 24 h after immunization and then gradually recovered during the following 1–2 weeks (Fig. 3b). This may indicate that LYM egressed from the circulation and entered tissues or secondary lymphoid organs. GRAN and in some cases WBC and MONO showed an increase at 24 h, which may be due to the inflammatory response caused by vaccination. Most of these parameters returned to baseline levels after 1–2 weeks. We also observed increases in PLT and MPV after 1–2 weeks (Fig. 3b). On the contrary, there was a decrease and then gradual recovery of RBC, HCT and HGB over 1–2 weeks, which may be an effect of the repetitive blood collection within a short period. Other erythrocyte-related parameters did not show substantial changes (Fig. 3c). The values of a few parameters transiently exceeded the identified RIs at 24 h, but the vast majority of data points remained within the RIs at 1–2 weeks postvaccination. Similarly, most of the biochemical and hematological parameters of Chinese rhesus macaques and cynomolgus macaques fluctuated within the identified RIs shortly after vaccination and returned to baseline levels after 1–2 weeks (Supplementary Figs. 3a–c and 4a–c). Some differences in the magnitude of changes in the parameters were observed between the studies, indicating that the type of vaccine has an impact on the early inflammatory response. In summary, most of the biochemical and hematological parameters of macaques returned to baseline levels within 1–2 weeks after vaccination.

We analyzed longitudinal data collected from Indian rhesus macaques that were used in three consecutive vaccine studies performed over a 3-year period. After each vaccine study, the animals underwent a washout period ranging from 3 months to 1 year before being included in the next study. Baseline samples were collected before each vaccination and analyzed for comparisons. The majority of the parameters remained at similar levels during this period and within the heathy ranges (Fig. 3d–f).

### Interleukin-1 signaling activation after vaccination

**Fig. 4:**
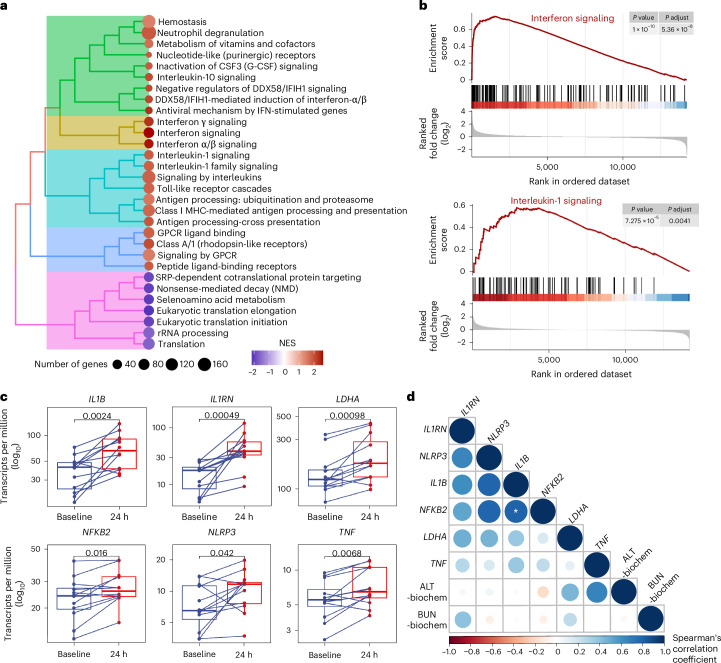
Blood transcriptomic analysis of Indian rhesus macaques before and 24 h after immunization. All the analyses are based on the same animals from a spike protein COVID-19 vaccine study^44^. Samples size *n* = 12. **a**, GSEA is based on the Reactome Pathway Database^51^. The plot is presented as a tree plot using hierarchical clustering, where closely related gene sets are grouped into the same clade according to the Jaccard similarity index of their genes. The radius of the circles represents the number of genes enriched in a given gene set. Upregulated genes are shown in red, and downregulated genes in blue, based on the normalized gene set enrichment score (NES). MHC, major histocompatibility complex. **b**, Interferon signaling and interleukin-1 signaling pathway enrichment analysis. The enrichment score is calculated using GSEA and is depicted as a running enrichment score curve, alongside the ranked list (log_2_ fold change) and the positions of pathway-associated genes within the ranked dataset. **c**, Box plots of transcripts of interleukin-1 signaling-related genes are presented in transcripts per million (log_10_) before and after immunization. Lines connect the paired data points. The box plots’ lower and upper hinges correspond to the 25th and 75th percentiles, while the upper and lower whiskers extend to 1.5 times the interquartile range. The line in the middle represents the median. The Wilcoxon statistical test for paired comparisons was performed, and *P* values are shown in the figure. **d**, The Spearman’s correlation shows the 24-h post-immunization fold change in interleukin-1 signaling-related genes (*IL1B*, *IL1RN*, *LDHA*, *NFKB2*, *NLRP3* and *TNF*) and biochemical parameters (ALT and BUN). Circle radii are scaled on the basis of the absolute value of Spearman’s correlation coefficient. Stars were added to indicate significant correlations, determined after FDR adjustment at a significance level of *P* < 0.05. All statistical comparisons are adjusted using the Benjamini–Hochberg procedure; adjusted *P* values are shown. An adjusted *P* < 0.05 is considered significant. Biochem, biochemical.

Previous studies have suggested a significant role of pro-inflammatory cytokines, such as IL-1β and IL-18, in liver inflammation and damage^48–50^. In addition, genes related to the interleukin-1 signaling pathway have been highlighted in large-scale human vaccine studies^41,42^. Because we observed a transient vaccine-induced effect on the liver at 24 h, we examined whether modulation of inflammation-related genes could be observed simultaneously. We compared transcriptomic profiles from RNA sequencing of blood samples taken from Indian rhesus macaques before and 24 h after immunization with a coronavirus disease 2019 (COVID-19) protein vaccine^44^ alongside biochemical and hematological assessments. We performed gene set enrichment analysis (GSEA) to understand which groups of genes became more active or less active in response to vaccination. The results were visualized in a tree plot, which organizes gene sets or pathways into clusters on the basis of their degree of overlapping genes, using the Reactome Pathway Database^51^. We found that changes in gene expression detected in blood 24 h after immunization were primarily associated with processes such as neutrophil degranulation, antigen processing and presentation and Toll-like receptor cascades (Fig. 4a). Notably, the interferon and interleukin-1 signaling pathways were among the most substantially upregulated pathways in the analysis. As shown in the GSEA plot, the enrichment score curve peaks sharply near the top of the ranked gene list, indicating that many genes associated with these pathways are among the most highly upregulated based on fold-change rankings. This observation is further supported by statistically significant adjusted *P* values, confirming the strong enrichment of pathway-related genes within the overall gene set (Fig. 4b). Transcripts of genes before and after immunization showed upregulation of *IL1B* and its upstream related genes *LDHA*, *NFKB2* and *NLRP3* in the interleukin-1 signaling pathway, along with downstream activated *TNF* and antagonistic *IL1RN* genes (Fig. 4c). Genes associated with another inflammasome-related cytokine gene, *IL18*, and the noncanonical inflammasome pathway, such as the caspase series genes *CASP4*, *CASP5*, *CASP7* and *CASP8*, were also activated 24 h after immunization (Supplementary Fig. 5a). Altogether, these results show that interleukin-1 signaling was activated after immunization, accompanied by upregulation of liver enzymes or metabolites. However, no direct correlation was identified between the 24-h post-immunization fold change in interleukin-1 signaling-related genes (*IL1B*, *IL1RN*, *LDHA*, *NFKB2*, *NLRP3* and *TNF*) and the fold change in parameters such as ALT and BUN (Fig. 4d).

### Monocyte activation after vaccination

**Fig. 5:**
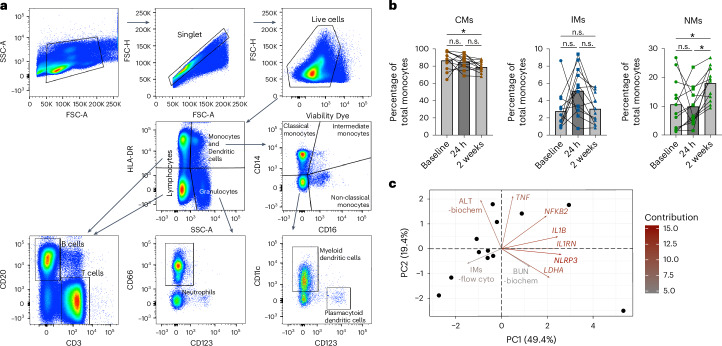
Monocyte subset dynamics and PCA after immunization. All the analyses are based on the same Indian rhesus macaques from a spike protein COVID-19 vaccine study^44^. Sample size *n* = 12. **a**, Flow cytometry gating strategy used to analyze cell frequencies and phenotypic profiles of NHPs from PBMCs. Representative PBMCs from rhesus macaques are shown in the figure. FCS, forward scatter; SSC, side scatter. **b**, The proportion of CMs, IMs and NMs in total monocytes, showing the changes in monocyte subsets at baseline, 24 h and 2 weeks after immunization. Bars indicate mean values. **c**, The PCA analysis of the 24-h post-immunization fold change in biochemical parameters (ALT and BUN), IMs and interleukin-1 signaling-related genes (*IL1B*, *IL1RN*, *LDHA*, *NFKB2*, *NLRP3* and *TNF*) is shown as a biplot with samples (dots) and variables (arrows), with the arrows colored according to the contribution of each variable. The direction and length of each arrow indicate the influence of each variable on the principal components (PCs). A Friedman test with Dunn’s test corrected for multiple comparisons was used. *P < 0.05. n.s., not significant. Flow cyto, flow cytometry.

We also analyzed cell fluctuation and differentiation. To do this, we designed a comprehensive staining panel for flow cytometry (Table 5) to evaluate multiple immune cell subsets such as CD14^+^CD16^−^ classical monocytes (CMs), CD14^+^CD16^+^ intermediate monocytes (IMs) and CD14^−^CD16^+^ non-classical monocytes (NMs), myeloid dendritic cells (MDCs) and plasmacytoid dendritic cells (PDCs), neutrophils, B cells and T cells (Fig. 5a). Staining antibodies with cross-reactivity to rhesus macaque and cynomolgus macaque cells were carefully selected and screened (Supplementary Fig. 5b).

**Table 5:** Flow cytometry panel of fluorescently labeled antibodies for investigating cell frequencies and phenotypic information

Marker	Color	Clone	Vendor	Catalog number
CD40	FITC	5C3	BioLegend	334306
CD80	BV421	L307.4	BD Biosciences	564160
CCR7	PE-Dazzle 594	G043H7	BioLegend	353236
CD123	PerCp-Cy5.5	7G3	BD Biosciences	558714
CD3	APC-Cy7	SP34-2	BD Biosciences	557757
CD66	APC	TET2	Miltenyi	130-118-539
CD70	BV786	Ki-24	BD Biosciences	565338
HLA-DR	BV650	L243	BioLegend	307650
CD11c	PE-Cy7	3.9	BioLegend	301608
CD16	AF700	3G8	BD Biosciences	560713
CD20	BV605	2H7	BioLegend	302334
CD14	BV510	M5E2	BioLegend	301842

The selected staining antibodies are cross-reactive with cells from human, rhesus macaque and cynomolgus macaque.

We and many other groups have previously demonstrated that innate immune activation after vaccination is often associated with the appearance of IMs in the blood, both in humans^52,53^ and in NHPs^54,55^. An increase in the inflammatory monocyte subset, IMs, was observed at 24 h in Indian rhesus macaques, although this increase was not statistically significant (Fig. 5b). Principal component analysis (PCA) of the 24-h post-immunization fold change in biochemical parameters (ALT and BUN), IMs and interleukin-1 signaling-related genes (*IL1B*, *IL1RN*, *LDHA*, *NFKB2*, *NLRP3* and *TNF*) suggested that the increased expression of these genes contributed to the differences at 24 h after immunization compared with baseline in some individuals but not all, while IMs exhibited a different pattern of change (Fig. 5c). Taken together, the increase in liver enzymes and metabolites after vaccination occurred simultaneously as induction of inflammation characterized by upregulation of genes in the interleukin-1 signaling pathway as well as increase in IMs.

### Vaccine-induced adverse effects are associated with detectable biochemical and hematological changes outside the RIs

**Fig. 6:**
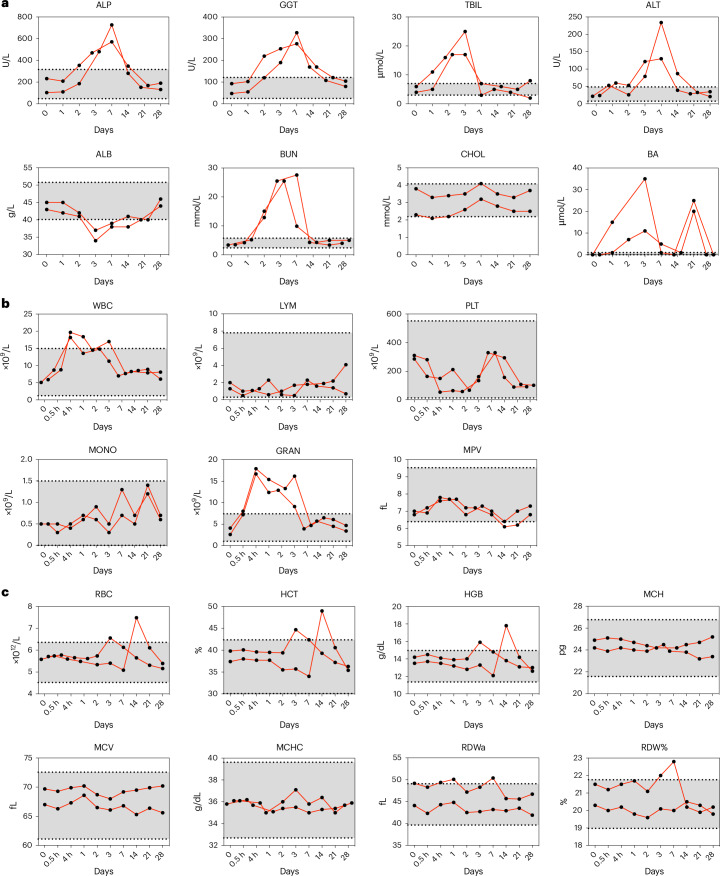
Biochemical and hematological changes over time in two Indian rhesus macaques exhibiting side effects after immunization^47^. Sample size *n* = 2. **a**–**c**, Results are shown as kinetics of biochemical parameters (baseline and days 1, 2, 3, 7, 14, 21 and 28) (**a**), leukocyte-related and thrombocyte-related (**b**), and erythrocyte-related (**c**) hematological parameters (baseline, 0.5 h, 4 h and days 1, 2, 3, 7, 14, 21 and 28). The dashed lines and gray areas indicate the identified Indian rhesus macaque RIs.

Because the animals analyzed in the study above showed no visible adverse effect and the changes in biochemical parameters as well as innate immune activation were rather modest, direct correlations were not concluded. We therefore analyzed in detail selected Indian rhesus macaques that had exhibited side effects after immunization. Among the several vaccine studies conducted in NHPs at KI, we have observed only two cases of severe adverse effects consisting of strong innate immune activation accompanied by temporary fever, vomiting and loss of appetite^47^. The adverse effects first appeared at day 1, showed substantial improvement by day 7 and were fully resolved by day 14. Biochemical parameters in this study were measured at baseline and on days 1, 2, 3, 7, 14, 21 and 28 (Fig. 6a). Hematological parameters were measured at the same time points plus additionally at 0.5 and 4 h (Fig. 6b,c). The two animals offered the opportunity to evaluate the association of side effects and biochemical and hematological values. We found that the most dramatic change in biochemical levels from prevaccination levels was observed 3–7 days after immunization rather than at 24 h in these two animals. Elevated levels of ALP, GGT, TBIL, ALT, BUN and BA were observed on day 3, while ALB reached its lowest level on the same day. By day 7, ALP, GGT, ALT and BUN levels had continued to rise to the peak levels detected (Fig. 6a). The kinetics of the hematological parameters were not always similar to the biochemical parameters. A substantial increase in WBC was observed as early as 4 h after immunization. This early increase in WBC consisted of mainly an increase in GRAN, while later at days 7–21 elevated levels of MONO were observed. Some hematological parameters fluctuated outside of the identified RIs, such as WBC, GRAN, MPV, RBC, HCT, HGB, RDWa and RDW%. However, all of them returned to normal levels within the RIs by day 28 (Fig. 6b,c).

**Fig. 7:**
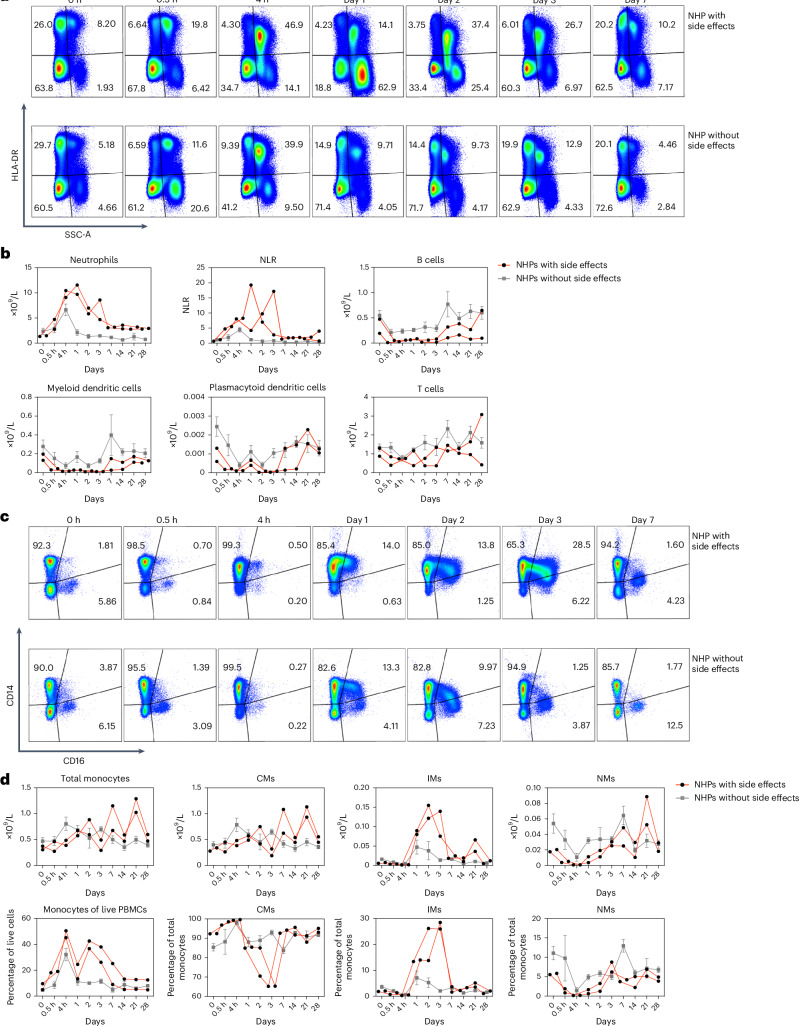
Characterization of Indian rhesus macaques exhibiting side effects^47^ using combined hematological and flow cytometry data. **a**, A comparison of cell frequencies over time using the same gating strategy as in Fig. 5a. Representative animals are shown. The numbers in the table represent the proportions. **b**, Normalized frequencies of cell subsets in NHPs with side effects (*n* = 2) and NHPs without side effects (*n* = 4) over time. Data are represented as mean ± s.e.m. **c**, A comparison of monocyte subsets over time using the same gating strategy as in Fig. 5a. Representative animals are shown. The numbers in the segments represent the proportions. **d**, Normalized frequencies of total monocytes and monocyte subsets in NHPs with side effects (*n* = 2) and NHPs without side effects (*n* = 4) over time. Data are represented as mean ± s.e.m.

The two animals experiencing adverse events were further investigated in terms of cellular responses. They were compared with other animals immunized simultaneously with a lower dose of the same vaccine formulation, which did not display any adverse effects. The same staining panel was used as described in Table 5 and Fig. 5a. We integrated hematological data from Fig. 6b with phenotypic data obtained by flow cytometry and analyzed differences between animals that did or did not exhibit side effects. The flow cytometry data showed that neutrophils in animals experiencing side effects rapidly increased at 0.5 h and peaked at 4–24 h and were sustained at elevated levels for at least 3 days, similar to what was detected for GRAN and WBC in the hematological analyses (Figs. 6b and 7a,b). The animals without side effects also showed a rapid increase in neutrophils, but the levels did not reach as high levels and returned to baseline levels at 24 h (Fig. 7a,b). The two animals experiencing side effects also showed an increase in the neutrophil-to-lymphocyte ratio (NLR) at 0.5–72 h (day 3). This period corresponded largely to the period when the animals exhibited visible side effects. By contrast, the animals without side effects had only a slight increase at 4 h (Fig. 7b). B cells, MDCs and PDCs decreased in both groups early after vaccination but recovered at 7 days. T cells showed no substantial changes (Fig. 7b). However, it is important to note that the limited number of animals included in this analysis makes it difficult to draw conclusions regarding changes in specific cells or parameters.

Both animals with and without side effects exhibited a rapid differentiation of IMs on day 1 after immunization. The change to a lower proportion of CMs and higher of IMs persisted up to day 3 and were more pronounced in the animals experiencing side effects, compared with the shorter period in those that did not exhibit side effects (Fig. 7c,d). Both groups of animals showed a slight decrease of NMs, but they returned to baseline levels on days 3–7. The percentage of total monocytes out of live peripheral blood mononuclear cells (PBMCs) increased rapidly at 4 h, declined and then reappeared on days 2–3 in animals experiencing side effects, but not in those that did not exhibit side effects (Fig. 7c,d). The elevated levels of MONO in the hematological analysis were thus observed later (days 3–21) (Fig. 6b) than the levels found by flow cytometry of PBMCs. This is probably explained by the fact that the hematological analyses are performed in whole blood, where the vast majority of cells consist of granulocytes early after vaccination, while the flow cytometry analyses are performed in PBMCs, where many granulocytes are depleted in the Ficoll separation process.

## Discussion

Establishing RIs of biochemical and hematological parameters is crucial for investigating the toxicity and safety of vaccines and drugs and observing changes in immune cell composition in experimental subjects. In this study, we compared classical biochemical and hematological parameters in the PAD with rhesus and cynomolgus macaques housed at our NHP facility at KI. The parameters showed a great overlap in levels, but we identified some significant differences such as higher MONO levels across all three macaque types at KI. These discrepancies may be due to differences in the analytical methods and instrument calibrations rather than representing true biological differences between animals in the different facilities. However, the same sample set would need to be analyzed by different laboratories to formally rule this out. In addition, macaques showed species-specific differences compared with human RIs, such as higher ALP and ALT levels and lower CHOL levels, highlighting the need for species-specific considerations when interpreting laboratory results.

In human studies, population-specific RIs are primarily established on the basis of the EP28-A3c guideline^39^, which is the state-of-the-art approach. These guidelines can also be reasonably applied to macaque studies^56^. The RIs in most NHP studies were commonly sourced from human RIs or were not properly calculated in accordance with standardized guidelines^22–26^. In this study, we calculate RIs as per the EP28-A3c guideline, reflecting the local population characteristics of macaques housed at KI. For future applications, we recommend using RIs derived from larger sample sizes and validating them in local populations before use^39,57^.

Using identified RIs, we analyzed biochemical and hematological changes before and after vaccination, across multiple vaccinations and in animals with and without side effects. Vaccination revealed several significant changes in the biochemical and hematological parameters despite no visible side effects. For instance, ALT and BUN showed transient changes within 24 h but returned to baseline within 1–2 weeks. LYM decreased, while GRAN increased initially but gradually recovered within 1–2 weeks. These trends have also been observed in other studies^44,45,47,55^. Some parameters such as TBIL and BA showed unique trends in Chinese rhesus and cynomolgus macaques. These characteristic changes may partly reflect immune responses induced by different types of vaccines. Nevertheless, for all three types of macaque, the transient fluctuations in liver-related markers most often remained within identified RIs, meaning that no durable liver provocation was observed using the parameters analyzed. Longitudinal analysis showed that some parameters had trends related to age rather than immunization effects, and these mostly remained within the identified RIs. Animals with side effects exhibited substantial changes outside of the identified RIs in parameters such as ALP, GGT, TBIL, ALT, BUN, BA and ALB, as well as some hematological parameters. Notably, the increase in BUN was opposite to the trend observed in healthy animals shown in Fig. 3a.

In particular, changes in ALT and BUN levels could be used as possible biomarkers to assess toxicity and inflammation induced by vaccination. Drug metabolism occurs primarily in the liver^29,30^, while excretion mainly takes place in the kidneys^58,59^. ALT is a key indicator for assessing the presence of liver damage^60^. These cytoplasmic enzymes, predominantly released by damaged hepatocytes, leak into the systemic circulation, indicating specific damage to hepatocellular structures^29^. Vaccine-induced elevations in ALT are usually mild and transient. The liver has a strong capacity to recover, and once inflammation subsides, ALT levels typically return to normal^29^. In addition, transient elevations of ALT can physiologically occur in healthy individuals, both in humans and macaques, due to factors such as stress. These occurrences do not necessarily indicate pathological liver damage^60^.

BUN originates from the breakdown of proteins in the body, specifically from the conversion of ammonia to urea in the liver, which is then transported via the bloodstream to the kidneys for excretion^61^. The level of BUN can be an indicator of liver function, kidney function and overall protein metabolism^62,63^. Liver inflammation may reduce the liver’s ability to convert ammonia to urea, resulting in lower BUN levels^61,64^. Conversely, transient elevated BUN levels of animals with side effects in this study probably suggested renal inflammatory responses induced by antibody drugs^47,58,59,65^. Changes in BUN levels alone may not accurately reflect specific pathological conditions and need to be evaluated in combination with clinical symptoms^62,63^.

Using flow cytometry, we identified and characterized innate immune responses. We then integrated and normalized this information with hematological data to compare animals with and without side effects. In particular, neutrophils and monocyte differentiation showed distinct patterns in animals with side effects. It has been reported that the increase in neutrophils was substantially associated with acute liver inflammation and damage^66,67^. The distribution, function and behavior of neutrophils and lymphocytes can be regulated by stress hormones^68^; the ratio of neutrophils to lymphocytes in the peripheral blood has been suggested as an indirect measurement of animal discomfort^28,69^. Besides the inflammatory response induced by immunization, the higher NLR in animals with side effects may also suggest higher stress levels caused by these side effects. Moreover, measurement of monocyte differentiation has been reported to be a commonly reliable biomarker reflecting rapid activation of innate immunity^52–55^. Neutrophil and monocyte differentiation could therefore also represent readily accessible biomarkers for analyzing the tolerability of vaccines and treatments.

In addition, blood transcriptomic analysis highlighted upregulated interferon and interleukin-1 signaling pathways. The significance of the interferon signaling pathway has already been widely reported ^70–72^. Genes related to the interleukin-1 signaling pathway have also been described in large-scale human vaccine studies^41,42^ and the role of inflammasomes and IL-1β in liver inflammation and damage has been thoroughly discussed^48,73^. Inflammasomes triggers liver inflammation via pro-inflammatory cytokines such as IL-1β, resulting in a downstream cytokine cascade and sensitizing hepatocytes to cellular toxicity induced by TNF^48^. Blood-derived IL-1β can also activate inflammasome genes, such as *NLRP3* and *IL1B* in the liver by activating NF-κB, leading to more IL-1β cytokines released in the liver^73^. Furthermore, IL-1RA, encoded by *IL1RN* gene, acts as an antagonist of IL-1β in regulating inflammation, and the scale of IL-1RA induction differs among species^74^. Both rhesus and cynomolgus macaques have shown a rapid increase in IL-1RA levels after immunization in previous studies. Higher levels of IL-1RA in macaques could lead to lower IL-1β levels and, thus, more effective control of inflammation^74,75^. Our RNA sequencing data provided insights into potential liver toxicity or inflammatory-related pathways, but these still need to be further explored in future studies.

A limitation of this study is that we did not perform direct comparative transcriptomic analysis of liver samples between NHPs with and without side effects before and after immunization. Although upregulation of related genes has been reported in human studies^76–78^, future NHP studies are expected to address this gap.

In conclusion, this study emphasizes the importance of establishing appropriate RIs for macaques to improve the quality of data obtained from preclinical research. Our integrative approach provides a comprehensive framework for assessing immunization impacts, refining vaccine safety studies and improving the applicability of NHP models in biomedical research and drug development.

## Methods

### Animals

This study was approved by the Stockholm Regional Ethical Board on Animal Experiments. Approved animal protocol numbers: 18427-2019 with amendments 10895-2020, 20678-2021, 13458-2021 and 22175-2023 and N2/15 with amendments N193/16 and 2379-2017. A total of 77 Indian-derived rhesus macaques (41 males and 36 females), 36 Chinese-derived rhesus macaques (18 males and 18 females) and 22 cynomolgus macaques (10 males and 12 females) were housed at Astrid Fagraeus Laboratory, KI. All procedures were performed according to the guidelines of the Association for Assessment and Accreditation of Laboratory Animal Care.

Macaques were housed in conditions designed to promote their well-being and maintain natural social structures. They were kept in groups of two to ten individuals with established hierarchy in the presence of a dominant individual alongside subdominant members. The housing environment featured ample space for play, private resting areas and enrichment such as wooden structures, toys and water pools. In the cold season, all macaques were housed indoors while during the warm season some, but not all, macaques had access to outdoor areas. The animals were fed pellets in the morning and afternoon, with vegetables provided in the afternoon. Grain, nuts and raisins were regularly offered as enrichment to encourage natural foraging behavior. Water was provided ad libitum.

The NHPs tested negative for herpes B virus, simian immunodeficiency virus, simian retrovirus and simian T-lymphotropic virus by enzyme-linked immunosorbent assay, for *Mycobacterium tuberculosis* by skin test, for intestinal parasites by microscopy and for *Salmonella*, *Shigella* and *Yersinia* by cultivation both at the breeder and in the quarantine at the vendor in the Netherlands. The NHPs were also tested at arrival at KI with microscopy and PCR for *Entamoeba histolytica* and *Giardia intestinalis*. Yearly tests of these agents above were performed for the NHPs.

All animals underwent behavioral training to minimize stress during handling and to respond to commands for easier capture and sedation. The animals were sedated for blood sampling and vaccinations with Ketaminol vet. (100 mg/mL, MSD Animal Health) at a dose of 10–15 mg/kg administered intramuscularly.

### Safety, biochemical and hematological tests

For macaque-derived blood samples, hematological analyses of heparinized blood were performed within 8 h after collection on an Exigo Vet instrument (Model H400, Boule Diagnostics AB) after quality control (QC) with use of Boule Vet Con control blood. Heparinized plasma samples were analyzed using an ABAXIS Vetscan VS2 3.1.35 Chemistry analyzer (Triolab). Indicated parameters were analyzed on Mammalian Liver Profile rotors (Triolab), which have individual QC controls.

### PAD

This research was made possible using data obtained from the PAD (https://primatedatabase.org/), an initiative of the National Institute on Aging. All animals in the database are nonexperimental control animals and healthy at the time of measurement.

### Immunizations and sampling

This study includes four published protein vaccine studies^44–47^ on Indian rhesus macaques (Fig. 3a–f), two mRNA vaccine studies^43^ on Chinese rhesus macaques (Supplementary Fig. 3a–c), including one unpublished influenza mRNA vaccine, and two unpublished protein vaccine studies on cynomolgus macaques (Supplementary Fig. 4a–c) involving malaria protein in adjuvant or cancer peptides with anti-CD40 mAb. These studies were used to calculate RIs and assess parameter changes after immunization. An additional baseline (pre-immunization) dataset from an unpublished study was included for calculating RIs of Indian rhesus macaques. All animals received a functional vaccine, not a control. Detailed information is provided in Table 1 (refs. ^43–47^).

### Blood sample processing

Rhesus macaque and cynomolgus macaque PBMCs were isolated by Ficoll-Paque (GE Healthcare) density gradient centrifugation of blood samples at 2,200 rpm for 25 min with no brake or acceleration as described previously^47^. PBMCs were washed in phosphate-buffered saline (PBS) for subsequent experiments.

### Flow cytometry for assessment of cell fluctuation and phenotype of immune cells

Fresh rhesus macaque and cynomolgus macaque PBMCs were stained with LIVE/DEAD Fixable Blue Dead Cell Stain Kit (Invitrogen, L23105) at 4 °C for 5 min and subsequently incubated with FcR Blocking Reagent (Miltenyi Biotec, 130-059-901) at 4 °C for 5 min according to the manufacturer’s instructions. Samples were then surface-stained with a panel of fluorescently labeled antibodies (Table 5). After staining and washing, PBMCs were resuspended in 1% paraformaldehyde and acquired on an LSRFortessa flow cytometer (Fortessa, BD). Data analysis was performed using FlowJo v10.

### Bulk transcriptomics and RNA sequencing data analysis

An RNA sequencing dataset from whole blood of pre-immunized and 24 h post-immunized Indian rhesus macaques was used (BioProject: PRJNA975321)^44^. Preprocessing of samples was done using the nf-core RNAseq pipeline (v3.7), with genome alignment via STAR alignment (v2.7.10a) to the Macaca mulatta genome (Mmul_10) and quantification with Salmon (v1.8.0). A custom bioinformatic analysis was performed in R programming (v4.4.2), the code is available in GitHub (https://github.com/Lore-Lab-Vaccine-Immunology/nhp_reference). Differential gene expression analysis was performed using DESeq2 (v1.34.0), and GSEA was conducted with Reactome Pathway Database^51^ using msigdbr (v7.5.1) and clusterProfiler (v.4.6.2) packages and plotted using enrichplot (v1.18.4)^79^. Differentially expressed genes were compared using a Wald test, with multiple hypothesis testing controlling the false discovery rate (FDR) through the Benjamini–Hochberg procedure (*q* value <0.05). GSEA was conducted using the ranked log_2_ fold change calculated when estimating the differentially expressed genes through DESeq2. The selected individual genes were presented in transcripts per million, and differences were calculated with the Wilcoxon signed-rank test. A nonparametric Spearman’s correlation with FDR-adjusted *P* values was used for correlation calculation. The PCA analysis was shown as a biplot with samples (dots) and variables (arrows).

### Statistical analyses

The RIs of biochemical and hematological parameters were calculated in accordance with the EP28-A3c guideline^39^. The data points were initially compiled by applying the ROUT test (*Q* = 1%) to identify outliers, followed by a test for normal distribution. Depending on normality and sample size, two methods were used for analyzing the cleaned data: (A) If the data points were consistent with a normal distribution: upper limit = mean + 1.96 × s.d., lower limit = mean − 1.96 × s.d. (B) If the data points were not consistent with a normal distribution and the sample size was less than 120, the robust method described in the EP28-A3c guideline was used, using MedCalc (v22.023). This method combines parametric and nonparametric approaches, using robust measures of location and spread instead of the mean and standard deviation^39,40^. The calculated ranges of RIs for biochemical and hematological parameters, representing 95% of the data points, are reported in Table 4.

A Mann–Whitney *U* test was used for comparing two unpaired groups. A Kruskal–Wallis test with Dunn’s test corrected for multiple comparisons using statistical hypothesis testing was used for comparisons involving three or more unpaired groups. A Friedman test with Dunn’s test corrected for multiple comparisons was used for three or more paired groups. The two-tailed *P* value was used in all statistical analyses comparing the study groups. Results were considered statistically significant when *P < 0.05, **P < 0.01, ***P < 0.001, ****P < 0.0001. Analyses were performed using GraphPad Prism 10 and R programming (v4.4.2).

### Reporting summary

Further information on research design is available in the Nature Portfolio Reporting Summary linked to this article.

## Online content

Any methods, additional references, Nature Portfolio reporting summaries, source data, extended data, supplementary information, acknowledgements, peer review information; details of author contributions and competing interests; and statements of data and code availability are available at 10.1038/s41684-025-01547-y.

## Supplementary information

Supplementary Information Supplementary Figs. 1–5 and Table 1.

Reporting Summary

## Data Availability

The RNA sequencing dataset analyzed during the current study is available on NCBI BioProject (PRJNA975321)^44^. The code generated in this study is available via GitHub at https://github.com/Lore-Lab-Vaccine-Immunology/nhp_reference. The Primate Aging Database (https://primatedatabase.org/) was last accessed in March 2024. The data that support the findings of this study are available from the corresponding author upon request.
