# Supplementary material for: Biochemical and hematological reference intervals in rhesus and cynomolgus macaques and implications for vaccine and drug development
Source: Lab Anim (NY). 2025 May 16;54(6):141–55. doi: 10.1038/s41684-025-01547-y (PMC12129814; doi:10.1038/s41684-025-01547-y)
Supplement: Supplementary file 1 — Supplementary Figs. 1–5 and Table 1. [file 41684_2025_1547_MOESM1_ESM.pdf]

lab animal

Supplementary information

https://doi.org/10.1038/s41684-025-01547-y

Biochemical and hematological reference intervals in rhesus and cynomolgus macaques and implications for vaccine and drug development

In the format provided by the authors and unedited

Supplementary Figure 1

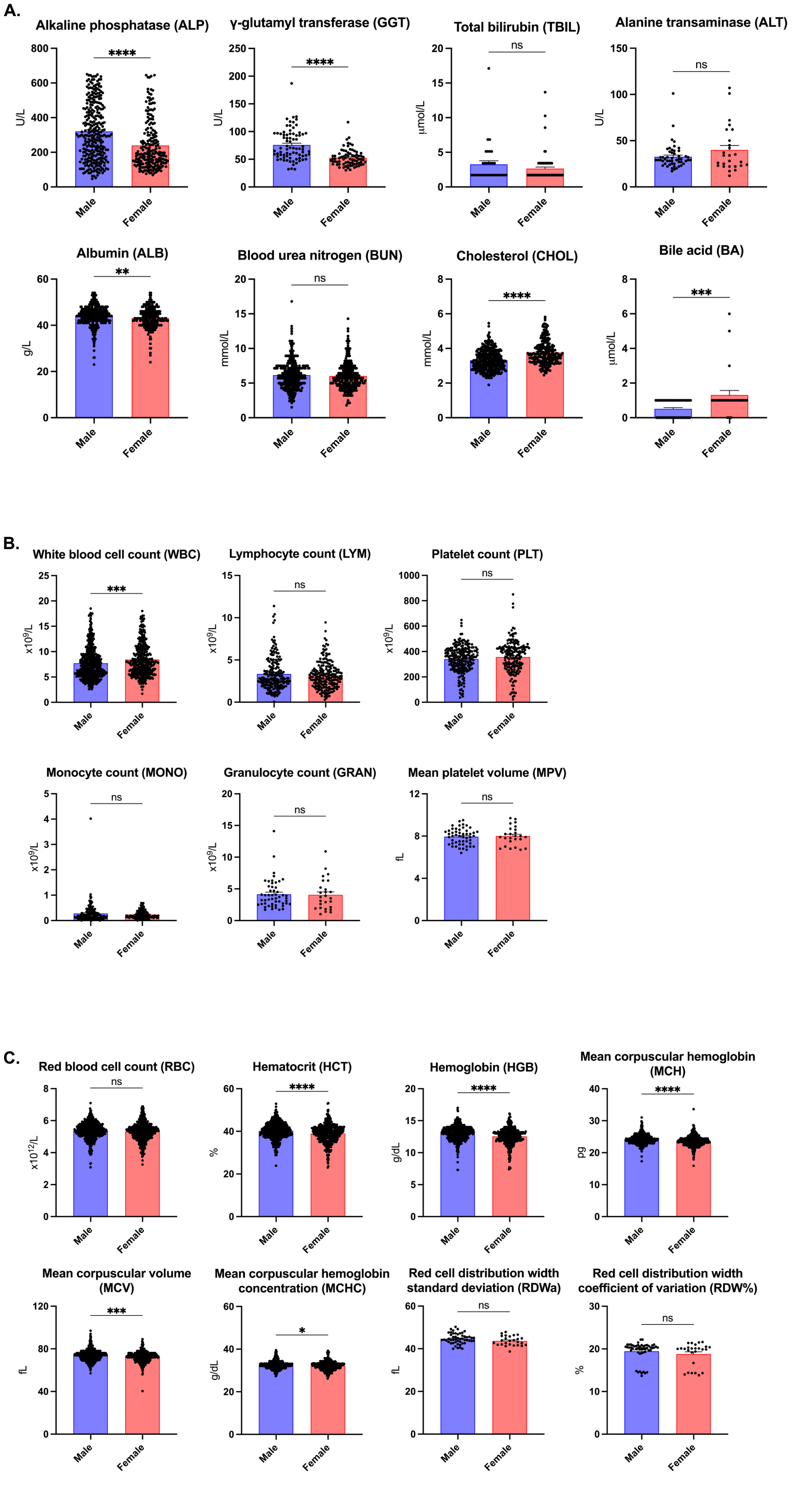

Supplementary Figure 1. Sex-based differences in biochemical and hematological parameters of Indian rhesus macaques housed at KI and the Young Adults (3-7 years) age group in the PAD database, if applicable. The sample size (n) ranged from 26 to 513 per group. **a,** Biochemical parameters. **b,** Leukocyte-related and thrombocyte-related parameters. Blue indicates male and red indicates female. **c,** Erythrocyte-related parameters. A Mann-Whitney U test was used. Results are considered statistically significant when $*P<0.05$, $**P<0.01$, $***P<0.001$, $****P<0.0001$. ns = not significant. mean ± SEM.

Supplementary Figure 2

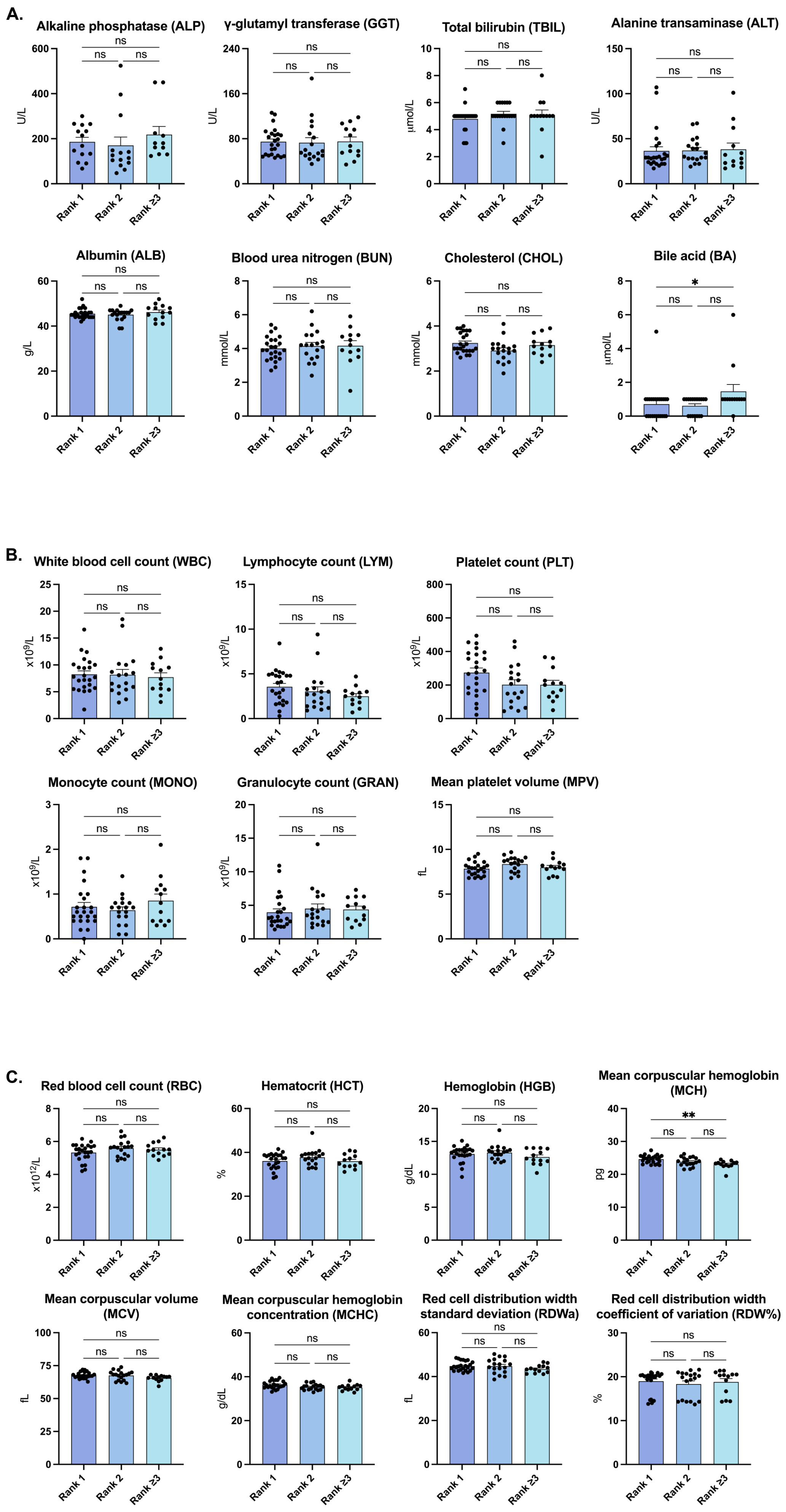

Supplementary Figure 2. Social hierarchy-based differences in biochemical and hematological parameters of Indian rhesus macaques housed at KI. The sample size (n) ranged from 11 to 24 per group. **a,** Biochemical parameters. **b,** Leukocyte-related and thrombocyte-related parameters. **c,** Erythrocyte-related parameters. Darker blue indicates higher rank. Rank 1 is the dominant animal, Rank 2 is the subdominant animal, and so on in each housing unit. A Kruskal-Wallis test with Dunn's test corrected for multiple comparisons using statistical hypothesis testing was used. Results are considered statistically significant when *$P<0.05$, **$P<0.01$. ns = not significant. mean ± SEM.

Supplementary Figure 3

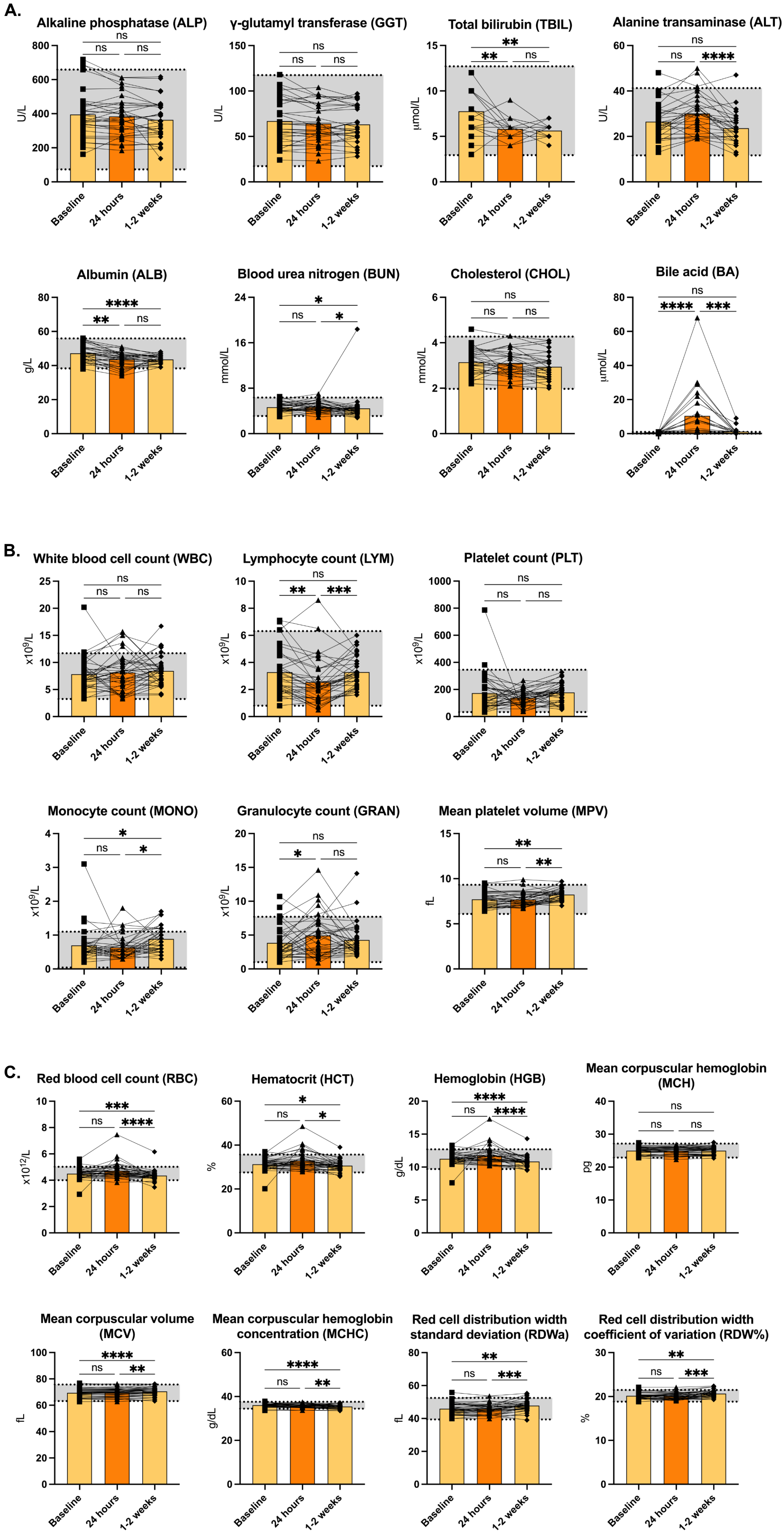

Supplementary Figure 3. Comparison of biochemical and hematological parameters in Chinese rhesus macaques before and after immunization in two mRNA vaccine studies (including one unpublished) listed in Table 1. The sample size (n) ranged from 32 to 36 per group. Peripheral blood samples were taken from Chinese rhesus macaques at baseline, 24 hours, and 1-2 weeks after immunization. **a,** Biochemical parameters. **b,** Leukocyte-related and thrombocyte-related parameters. **c,** Erythrocyte-related parameters. Symbols (squares, triangles, and diamonds) represent data points from different time points from the same animals, and the bars indicate the mean values. Dashed lines and grey areas indicate the identified Chinese rhesus macaque reference intervals (RIs). A Friedman test with Dunn's test corrected for multiple comparisons was used. Results are considered statistically significant when *P<0.05, **P<0.01, ***P<0.001, ****P<0.0001. ns = not significant.

Supplementary Figure 4

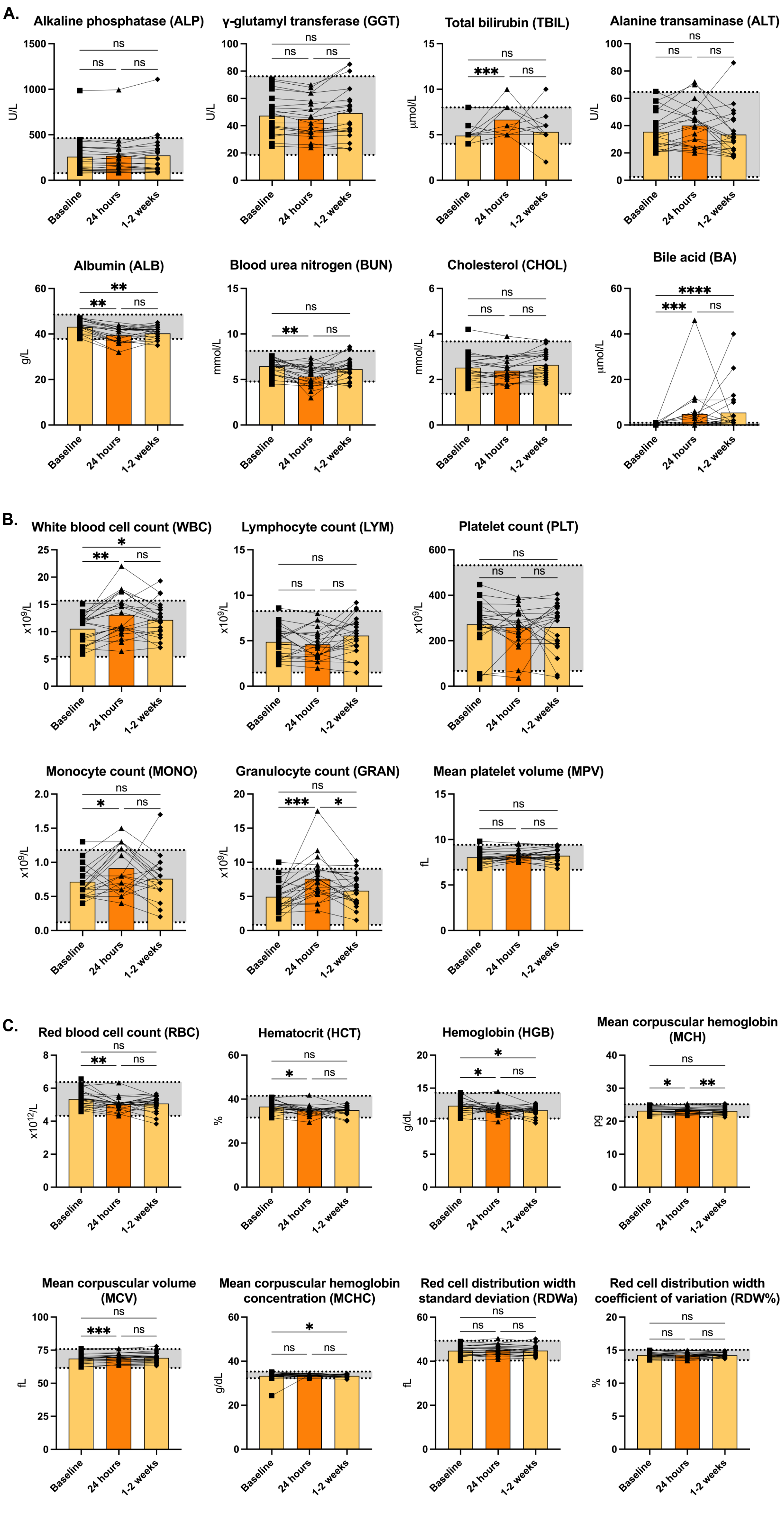

Supplementary Figure 4. Comparison of biochemical and hematological parameters in cynomolgus macaques before and after immunization in two unpublished protein vaccine studies listed in Table 1. n=22. Peripheral blood samples were taken from cynomolgus macaques at baseline, 24 hours, and 1-2 weeks after immunization. **a,** Biochemical parameters. **b,** Leukocyte-related and thrombocyte-related parameters. **c,** Erythrocyte-related parameters. Symbols (squares, triangles, and diamonds) represent data points from different time points from the same animals, and the bars indicate the mean values. Dashed lines and grey areas indicate the identified cynomolgus macaque reference intervals (RIs). A Friedman test with Dunn's test corrected for multiple comparisons was used. Results are considered statistically significant when *P<0.05, **P<0.01, ***P<0.001, ****P<0.0001. ns = not significant.

Supplementary Figure 5

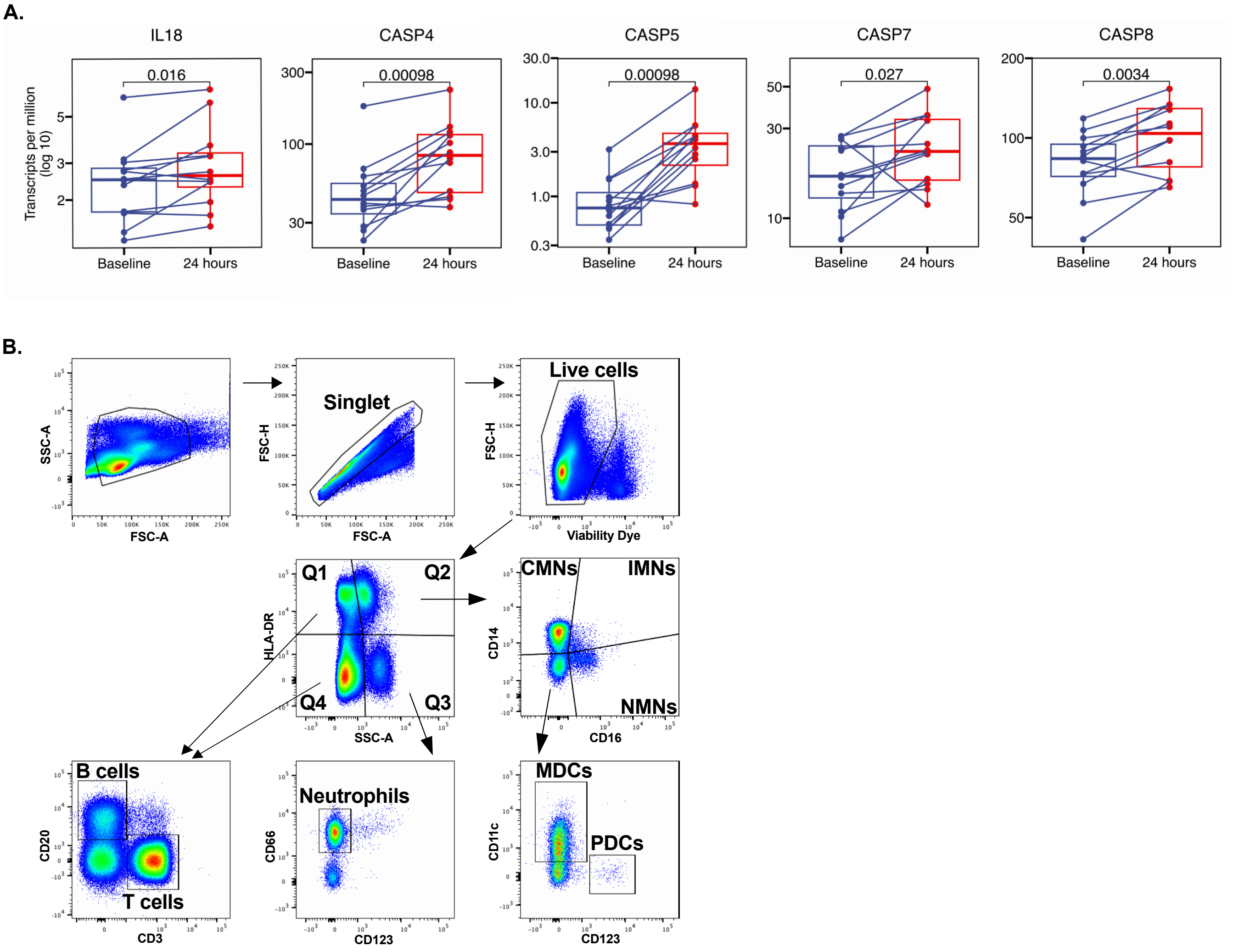

Supplementary Figure 5. Transcripts of selected genes and flow cytometry gating strategy. a, Boxplots of transcripts of inflammasome-related cytokine gene (*IL18*) and genes associated with the non-canonical inflammasome pathway (*CASP4*, *CASP5*, *CASP7*, and *CASP8*) are presented in transcripts per million (log10) before and after immunization. Lines connect the paired data points. The boxplots' lower and upper hinges correspond to the 25th and 75th percentiles, while the upper and lower whiskers extend to 1.5 times the interquartile range. The line in the middle represents the median. The Wilcoxon statistical test for paired comparisons was performed, and *p*-values are shown in the figure. Peripheral blood samples were taken from Indian rhesus macaques pre- and 24 hours post-immunization. n=12. **b,** Flow cytometry gating strategy for investigating the cell frequencies and phenotypic information of NHPs. Representative cynomolgus macaque PBMCs are shown.

Supplementary Table 1

Species	Age group		Parameter	Number of values	Mean	Std. Deviation	Unit
**Indian *Macaca mulatta***	Infants/juveniles (<3 years)	Biochemical	ALP	115	502.58	107.56	U/L
			ALT	N/A	N/A	N/A	U/L
			GGT	115	70.00	21.18	U/L
			BA	N/A	N/A	N/A	μmol/L
			TBIL	97	3.12	1.18	μmol/L
			ALB	190	42.96	4.59	g/L
			BUN	216	6.70	1.70	mmol/L
			CHOL	174	3.94	0.66	mmol/L
		Hematological	RBC	479	5.34	0.64	$x10^{12}$/L
			WBC	455	8.89	3.25	$x10^{9}$/L
			PLT	365	393.93	116.27	$x10^{9}$/L
			GRAN	N/A	N/A	N/A	$x10^{9}$/L
			LYM	278	5.04	2.43	$x10^{9}$/L
			MONO	222	0.23	0.22	$x10^{9}$/L
			HCT	552	39.21	4.31	%
			HGB	544	12.32	1.52	g/dL
			MCH	473	22.87	1.86	pg
			MCV	419	70.08	4.60	fL
			MCHC	534	31.76	1.95	g/dL
			RDWa	N/A	N/A	N/A	fL
			RDW%	N/A	N/A	N/A	%
			MPV	N/A	N/A	N/A	fL
	Adults (7-12 years)	Biochemical	ALP	774	132.69	55.41	U/L
			ALT	N/A	N/A	N/A	U/L
			GGT	172	51.97	18.03	U/L
			BA	N/A	N/A	N/A	μmol/L
			TBIL	185	3.82	2.52	μmol/L
			ALB	744	41.66	4.72	g/L
			BUN	895	6.01	2.05	mmol/L
			CHOL	701	3.54	0.75	mmol/L
		Hematological	RBC	1938	5.43	0.60	$x10^{12}$/L
			WBC	1894	7.67	2.92	$x10^{9}$/L
			PLT	804	374.05	107.25	$x10^{9}$/L
			GRAN	N/A	N/A	N/A	$x10^{9}$/L
			LYM	1144	3.11	1.53	$x10^{9}$/L
			MONO	971	0.24	0.20	$x10^{9}$/L
			HCT	1919	40.29	4.01	%
			HGB	1889	12.92	1.32	g/dL
			MCH	1932	23.94	1.54	pg
			MCV	1698	74.56	4.35	fL
			MCHC	1927	32.15	1.49	g/dL
			RDWa	N/A	N/A	N/A	fL
			RDW%	N/A	N/A	N/A	%
			MPV	N/A	N/A	N/A	fL
	Elderly (≥17 years)	Biochemical	ALP	3894	140.28	80.15	U/L
			ALT	N/A	N/A	N/A	U/L
			GGT	2604	54.02	18.57	U/L
			BA	N/A	N/A	N/A	μmol/L
			TBIL	2587	3.70	2.39	μmol/L
			ALB	3497	37.37	4.24	g/L
			BUN	3939	6.15	1.93	mmol/L
			CHOL	3091	3.76	0.78	mmol/L
		Hematological	RBC	5671	5.88	0.69	$x10^{12}$/L
			WBC	5824	6.82	2.76	$x10^{9}$/L
			PLT	3518	328.58	100.07	$x10^{9}$/L
			GRAN	N/A	N/A	N/A	$x10^{9}$/L
			LYM	3999	2.72	1.35	$x10^{9}$/L
			MONO	3290	0.33	0.23	$x10^{9}$/L
			HCT	5795	42.90	4.75	%
			HGB	5775	13.82	1.59	g/dL
			MCH	5811	23.53	1.71	pg
			MCV	4783	73.06	4.94	fL
			MCHC	5811	32.27	1.28	g/dL
			RDWa	N/A	N/A	N/A	fL
			RDW%	N/A	N/A	N/A	%
			MPV	N/A	N/A	N/A	fL

Age group		Parameter	Number of values	Mean	Std. Deviation	Unit
Young Adults (3-7 years)	Biochemical	ALP	466	295.25	159.53	U/L
		ALT	N/A	N/A	N/A	U/L
		GGT	76	51.55	13.53	U/L
		BA	N/A	N/A	N/A	μmol/L
		TBIL	105	2.83	2.33	μmol/L
		ALB	518	43.42	4.59	g/L
		BUN	562	6.39	1.91	mmol/L
		CHOL	499	3.54	0.68	mmol/L
	Hematological	RBC	824	5.34	0.56	$x10^{12}$/L
		WBC	806	7.96	3.13	$x10^{9}$/L
		PLT	320	363.21	105.27	$x10^{9}$/L
		GRAN	N/A	N/A	N/A	$x10^{9}$/L
		LYM	315	3.12	1.62	$x10^{9}$/L
		MONO	294	0.25	0.29	$x10^{9}$/L
		HCT	853	39.96	4.05	%
		HGB	833	12.80	1.24	g/dL
		MCH	808	23.95	1.65	pg
		MCV	730	74.03	4.92	fL
		MCHC	838	32.11	1.57	g/dL
		RDWa	N/A	N/A	N/A	fL
		RDW%	N/A	N/A	N/A	%
		MPV	N/A	N/A	N/A	fL
Middle-Aged (12-17 years)	Biochemical	ALP	1032	129.24	69.06	U/L
		ALT	N/A	N/A	N/A	U/L
		GGT	530	51.77	16.75	U/L
		BA	N/A	N/A	N/A	μmol/L
		TBIL	557	3.35	2.11	μmol/L
		ALB	993	39.47	4.47	g/L
		BUN	1085	5.72	1.98	mmol/L
		CHOL	785	3.82	0.88	mmol/L
	Hematological	RBC	1892	5.49	0.68	$x10^{12}$/L
		WBC	1882	7.14	2.79	$x10^{9}$/L
		PLT	1089	356.63	107.42	$x10^{9}$/L
		GRAN	N/A	N/A	N/A	$x10^{9}$/L
		LYM	1172	2.64	1.36	$x10^{9}$/L
		MONO	998	0.27	0.21	$x10^{9}$/L
		HCT	1903	40.54	4.60	%
		HGB	1878	13.10	1.51	g/dL
		MCH	1935	24.01	1.53	pg
		MCV	1758	74.12	4.37	fL
		MCHC	1935	32.43	1.44	g/dL
		RDWa	N/A	N/A	N/A	fL
		RDW%	N/A	N/A	N/A	%
		MPV	N/A	N/A	N/A	fL

Supplementary Table 1. Calculated means and standard deviations (SDs) of biochemical and hematological parameters across different age groups. Datasets of healthy Indian rhesus macaques in captivity in the PAD were stratified into 5 groups: Infants/juveniles (<3 years), Young Adults (3-7 years), Adults (7-12 years), Middle-Aged (12-17 years), and Elderly (≥17 years). The sample size (n) ranged from 76 to 5824 per group.
