# Supplementary material for: Biochemical and hematological reference intervals in rhesus and cynomolgus macaques and implications for vaccine and drug development
Source: Lab Anim (NY). 2025 May 16;54(6):141–55. doi: 10.1038/s41684-025-01547-y (PMC12129814; doi:10.1038/s41684-025-01547-y)
Supplement: Supplementary file 2 — Reporting Summary [file 41684_2025_1547_MOESM2_ESM.pdf]

nature portfolio

Corresponding author(s): Karin Loré

Last updated by author(s): Mar 20, 2025

Reporting Summary

Nature Portfolio wishes to improve the reproducibility of the work that we publish. This form provides structure for consistency and transparency in reporting. For further information on Nature Portfolio policies, see our Editorial Policies and the Editorial Policy Checklist.

Statistics

For all statistical analyses, confirm that the following items are present in the figure legend, table legend, main text, or Methods section.

n/a	Confirmed	
☐	☒	The exact sample size (n) for each experimental group/condition, given as a discrete number and unit of measurement
☐	☒	A statement on whether measurements were taken from distinct samples or whether the same sample was measured repeatedly
☐	☒	The statistical test(s) used AND whether they are one- or two-sided<br>*Only common tests should be described solely by name; describe more complex techniques in the Methods section.*
☐	☒	A description of all covariates tested
☐	☒	A description of any assumptions or corrections, such as tests of normality and adjustment for multiple comparisons
☐	☒	A full description of the statistical parameters including central tendency (e.g. means) or other basic estimates (e.g. regression coefficient) AND variation (e.g. standard deviation) or associated estimates of uncertainty (e.g. confidence intervals)
☒	☐	For null hypothesis testing, the test statistic (e.g. F, t, r) with confidence intervals, effect sizes, degrees of freedom and P value noted<br>*Give P values as exact values whenever suitable.*
☒	☐	For Bayesian analysis, information on the choice of priors and Markov chain Monte Carlo settings
☒	☐	For hierarchical and complex designs, identification of the appropriate level for tests and full reporting of outcomes
☒	☐	Estimates of effect sizes (e.g. Cohen's d, Pearson's r), indicating how they were calculated

Our web collection on statistics for biologists contains articles on many of the points above.

Software and code

Policy information about availability of computer code

Data collection	For macaques-derived blood samples, hematological analyses of heparinized blood were performed within 8 hours after collection on an Exigo Vet instrument (Model H400, Boule Diagnostics AB, Spånga, Sweden) after QC with use of Boule Vet Con control blood. Heparinized plasma samples were analyzed using an ABAXIS Vetscan VS2 3.1.35 Chemistry analyzer (Triolab, Solna, Sweden). Indicated parameters were analyzed on Mammalian Liver Profile rotors (Triolab), which have individual QC controls. The Primate Aging Database (https://primatedatabase.org/) was last accessed in March 2024. Flow cytometry data was collected using BD FACSDiva software (v9).
Data analysis	A custom bioinformatic analysis was performed in R, the code generated in this study is available on the GitHub repository (https://github.com/Lore-Lab-Vaccine-Immunology/nhp_reference) (v4.2.2). Flow data analysis was performed using FlowJo v10. Statistical analyses were performed using GraphPad Prism (v10) and MedCalc (v22.023).

For manuscripts utilizing custom algorithms or software that are central to the research but not yet described in published literature, software must be made available to editors and reviewers. We strongly encourage code deposition in a community repository (e.g. GitHub). See the Nature Portfolio guidelines for submitting code & software for further information.

Data

Policy information about availability of data

All manuscripts must include a data availability statement. This statement should provide the following information, where applicable:

- Accession codes, unique identifiers, or web links for publicly available datasets
- A description of any restrictions on data availability
- For clinical datasets or third party data, please ensure that the statement adheres to our policy

The RNA-Seq dataset analyzed during the current study is available on NCBI BioProject (PRJNA975321). The code generated in this study is available on the GitHub repository (https://github.com/Lore-Lab-Vaccine-Immunology/nhp_reference). The Primate Aging Database (https://primatedatabase.org/) was last accessed in March 2024. Data are available upon reasonable request to the corresponding author.

Research involving human participants, their data, or biological material

Policy information about studies with human participants or human data. See also policy information about sex, gender (identity/presentation), and sexual orientation and race, ethnicity and racism.

Reporting on sex and gender	N/A
Reporting on race, ethnicity, or other socially relevant groupings	N/A
Population characteristics	N/A
Recruitment	N/A
Ethics oversight	N/A

Note that full information on the approval of the study protocol must also be provided in the manuscript.

Field-specific reporting

Please select the one below that is the best fit for your research. If you are not sure, read the appropriate sections before making your selection.

☒ Life sciences ☐ Behavioural & social sciences ☐ Ecological, evolutionary & environmental sciences

For a reference copy of the document with all sections, see nature.com/documents/nr-reporting-summary-flat.pdf

Life sciences study design

All studies must disclose on these points even when the disclosure is negative.

Sample size	The sample size was based on the number of animals and data points available at both Karolinska Institutet and the Primate Aging Database.
Data exclusions	When calculating the reference intervals, the data points were initially compiled by applying the ROUT test (Q = 1%) to identify and clean up outliers in accordance with the EP28-A3c guideline.
Replication	Due to ethical and financial constraints, non-human primate studies were not replicated in identical studies. However, extensive data from multiple studies conducted at Karolinska Institutet, along with data from the Primate Aging Database, have been thoroughly analyzed to strengthen the findings.
Randomization	The animals used in each vaccine study were not randomly assigned to groups. Grouping was controlled by ensuring a similar distribution of weight and sex, when applicable.
Blinding	The data collection and analysis were not blinded, as we processed all available data points to the best of our ability.

Reporting for specific materials, systems and methods

We require information from authors about some types of materials, experimental systems and methods used in many studies. Here, indicate whether each material, system or method listed is relevant to your study. If you are not sure if a list item applies to your research, read the appropriate section before selecting a response.

Materials & experimental systems

n/a	Involved in the study
☐	☒ Antibodies
☒	☐ Eukaryotic cell lines
☒	☐ Palaeontology and archaeology
☐	☒ Animals and other organisms
☒	☐ Clinical data
☒	☐ Dual use research of concern
☒	☐ Plants

Methods

n/a	Involved in the study
☒	☐ ChIP-seq
☐	☒ Flow cytometry
☒	☐ MRI-based neuroimaging

Antibodies

Antibodies used	Information about the antibodies used in the flow cytometry panel is described in Table 5.
Validation	Species-specific reactivity and validation of all the antibodies used in this study were based on the manufacturer's reports and information on their official website.

Animals and other research organisms

Policy information about studies involving animals; ARRIVE guidelines recommended for reporting animal research, and Sex and Gender in Research

Laboratory animals	A total of 77 Indian-derived rhesus macaques (41 males and 36 females), 36 Chinese-derived rhesus macaques (18 males and 18 females), and 22 cynomolgus macaques (10 males and 12 females) housed at Karolinska Institutet were involved in this study. Animal datasets were also collected from the Primate Aging Database.
Wild animals	No wild animals were involved.
Reporting on sex	A sex-based comparison was performed (Supplementary Fig. 1) for the biochemical and hematological parameters of Indian rhesus macaques housed at KI and the Young Adults (3-7 years) age group in the PAD database, if applicable.
Field-collected samples	No field-collected samples were involved.
Ethics oversight	This study was approved by the Stockholm Regional Ethical Board on Animal Experiments (Approved animal protocol numbers: 18427-2019 with amendments 10895-2020, 20678-2021, 13458-2021, and 22175-2023 and N2/15 with amendments N193/16 and 2379-2017). All procedures were performed according to the guidelines of the Association for Assessment and Accreditation of Laboratory Animal Care.

Note that full information on the approval of the study protocol must also be provided in the manuscript.

Plants

Seed stocks	*Report on the source of all seed stocks or other plant material used. If applicable, state the seed stock centre and catalogue number. If plant specimens were collected from the field, describe the collection location, date and sampling procedures.*
Novel plant genotypes	*Describe the methods by which all novel plant genotypes were produced. This includes those generated by transgenic approaches, gene editing, chemical/radiation-based mutagenesis and hybridization. For transgenic lines, describe the transformation method, the number of independent lines analyzed and the generation upon which experiments were performed. For gene-edited lines, describe the editor used, the endogenous sequence targeted for editing, the targeting guide RNA sequence (if applicable) and how the editor was applied.*
Authentication	*Describe any authentication procedures for each seed stock used or novel genotype generated. Describe any experiments used to assess the effect of a mutation and, where applicable, how potential secondary effects (e.g. second site T-DNA insertions, mosiacism, off-target gene editing) were examined.*

Flow Cytometry

Plots

Confirm that:

☒ The axis labels state the marker and fluorochrome used (e.g. CD4-FITC).

☒ The axis scales are clearly visible. Include numbers along axes only for bottom left plot of group (a 'group' is an analysis of identical markers).

☒ All plots are contour plots with outliers or pseudocolor plots.

☒ A numerical value for number of cells or percentage (with statistics) is provided.

Methodology

Sample preparation	Rhesus macaque and cynomolgus macaque PBMCs were isolated by Ficoll-Paque (GE Healthcare, Fairfield, CT) density gradient centrifugation of blood samples at 2200 rpm for 25 minutes with no brake or acceleration. PBMCs were washed in phosphate-buffered saline (PBS) and ready for next experiment.
Instrument	PBMCs were acquired on an LSRFortessa flow cytometer (Fortessa, BD).
Software	Flow cytometry data was collected using BD FACSDiva software (v9). Data analysis was performed using FlowJo v10.
Cell population abundance	PBMCs were isolated by Ficoll-Paque (GE Healthcare, Fairfield, CT) density gradient centrifugation of blood samples at 2200 rpm for 25 minutes with no brake or acceleration.
Gating strategy	Gating strategy used to gate multiple immune cell subsets, such as CD14+CD16- classical monocytes (CMs), CD14+CD16+ intermediate monocytes (IMs), CD14-CD16+ non-classical monocytes (NMs), myeloid dendritic cells (MDCs), plasmacytoid dendritic cells (PDCs), neutrophils, B cells, and T cells, is shown in Fig. 5a and Supplementary Fig. 5b.

☒ Tick this box to confirm that a figure exemplifying the gating strategy is provided in the Supplementary Information.
